# EGF receptor kinase suppresses ciliogenesis through activation of USP8 deubiquitinase

**DOI:** 10.1038/s41467-018-03117-y

**Published:** 2018-02-22

**Authors:** Kousuke Kasahara, Hiromasa Aoki, Tohru Kiyono, Shujie Wang, Harumi Kagiwada, Mizuki Yuge, Toshio Tanaka, Yuhei Nishimura, Akira Mizoguchi, Naoki Goshima, Masaki Inagaki

**Affiliations:** 1 Department of Physiology, Mie University Graduate School of Medicine, Tsu, Mie 14101 Japan; 2 Department of Clinical Pharmacy, Graduate School of Pharmaceutical Sciences, Nagoya City University, Nagoya, Aichi 23903 Japan; 3 Division of Carcinogenesis and Cancer Prevention, National Cancer Center Research Institute, Tokyo, 82606 Japan; 4 Department of Neural Regeneration and Cell Communication, Mie University Graduate School of Medicine, Tsu, Mie 14101 Japan; 5 Molecular Profiling Research Center for Drug Discovery, National Institute of Advanced Industrial Science and Technology, Tokyo, 82626 Japan; 6 Department of Integrative Pharmacology, Mie University Graduate School of Medicine, Tsu, Mie 14101 Japan; 7 Department of Systems Pharmacology, Mie University Graduate School of Medicine, Tsu, Mie 14101 Japan

## Abstract

Ciliogenesis is generally inhibited in dividing cells, however, it has been unclear which signaling cascades regulate the phenomenon. Here, we report that epidermal growth factor receptor (EGFR) kinase suppresses ciliogenesis by directly phosphorylating the deubiquitinase USP8 on Tyr-717 and Tyr-810 in RPE1 cells. These phosphorylations elevate the deubiquitinase activity, which then stabilizes the trichoplein-Aurora A pathway, an inhibitory mechanism of ciliogenesis. EGFR knockdown and serum starvation result in ciliogenesis through downregulation of the USP8-trichoplein-Aurora A signal. Moreover, primary cilia abrogation, which is induced upon IFT20 or Cep164 depletion, ameliorates the cell cycle arrest of EGFR knockdown cells. The present data reveal that the EGFR-USP8-trichoplein-Aurora A axis is a critical signaling cascade that restricts ciliogenesis in dividing cells, and functions to facilitate cell proliferation. We further show that *usp8* knockout zebrafish develops ciliopathy-related phenotypes including cystic kidney, suggesting that USP8 is a regulator of ciliogenesis in vertebrates.

## Abstract (web summary)

The trichoplein-Aurora A pathway inhibits ciliogenesis in proliferating cells. Here the authors EGFR-mediated phosphorylation of the deubiquitinating enzyme USP8 leads to its activation, and this suppresses trichoplein degradation, allowing inhibition of ciliogenesis.

## Introduction

The primary cilia are microtubule-based sensory organelles that are grown from mother centrioles (also known as basal bodies) and protrude from the apical surface of quiescent cells. Primary cilia are considered to function as chemosensors and/or mechnosensors, and play critical roles in a variety of developmental signaling pathways^1–6^. Defects in ciliogenesis and dysregulated ciliary functions of this signaling antenna result in cell dysfunctions and multiple genetic diseases, collectively termed ciliopathies. These include polycystic kidney, microcephaly, retinal degeneration, situs inversus, and tumorigenesis^7–10^.

The presence of primary cilia has long been implicated in cell cycle progression: tissue culture cells generally form primary cilia when they are exposed to cell cycle exit signals such as serum starvation, and then serum stimulation induces primary cilia disassembly that is accompanied by cell cycle re-entry^11,12^. This mutually exclusive relationship between ciliogenesis and cell cycle progression is considered to allow centrosomes to duplicate and to function as the main microtubule-organizing centers and mitotic apparatuses in growing cells^3,6,13–17^. Recent studies have further revealed that primary cilia themselves drive the cell cycle checkpoint: delayed or defective primary cilia disassembly could block cell cycle re-entry upon serum stimulation of quiescent cells^18–23^, and conversely, loss of primary cilia accelerates the re-entry^24^. Moreover, when unscheduled ciliogenesis is induced by dysfunctions of negative cilia regulators, cells exit cell cycle even in growth conditions^23,25,26^. These observations suggest that several regulatory mechanisms coupled to cell cycle have evolved to ensure the timely onset of ciliognesis^13,14,16,17^.

We have previously shown that a centriolar protein, trichoplein, originally identified as a keratin-binding protein^27,28^, acts as a negative regulator of ciliogenesis in growing cells^25^. Trichoplein binds and activates Aurora A kinase especially at G1 phase, which then suppresses ciliogenesis. Knockdown of trichoplein or Aurora A causes unscheduled ciliogenesis-dependent cell cycle arrest in growth condition. Upon serum starvation-induced cell cycle exit, trichoplein is polyubiquitinated by the CRL3^KCTD17^ ubiquitin ligase and removed from the mother centriole through proteasome-mediated degradation, triggering Aurora A inactivation and ciliogenesis^23,26,29^. However, it remains unknown why trichoplein is resistant to degradation in growing cells because the CRL3^KCTD17^ functions are unchanged by serum starvation^26^.

In this study, we have sought to identify a deubiquitinase (DUB) that suppresses ciliogenesis by counteracting the CRL3^KCTD17^-mediated trichoplein degradation. Our small-interfering RNA (siRNA)-based functional screens identified six DUBs as negative regulators of ciliogenesis in RPE1 cells. Further analyses revealed that USP8 directly deubiquitinated trichoplein and stabilized its protein levels in growing cells. Most importantly, epidermal growth factor receptor (EGFR) kinase activated USP8 by phosphorylating Tyr-717 and Tyr-810. Therefore, serum starvation led to downregulation of the EGFR-USP8 signal, which allowed CRL3^KCTD17^ to target trichoplein for degradation, resulting in ciliogenesis. We further found that *usp8* knockout zebrafish developed ciliopathy-related anomalies, suggesting that USP8 functions as an important factor of ciliogenesis in vertebrates.

## Results

### The six DUBs function to suppress ciliogenesis

**Fig. 1:**
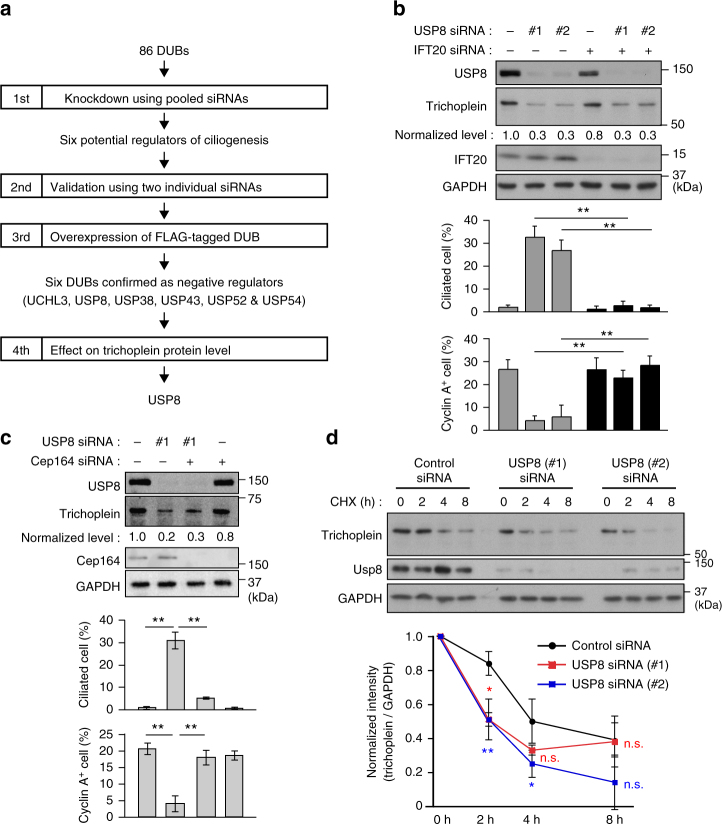
USP8 knockdown induces trichoplein degradation and unscheduled ciliogenesis. **a** Screen flowchart. **b**,** c** Twenty-four hours after transfection with siRNA for IFT20 (**b**) or Cep164 (**c**), PRE1 cells were further transfected with USP8 siRNA (#1 or #2) and then cultured for 48 h in the presence of serum (10% FBS). The cells were analyzed by immunoblotting with indicated antibodies. Normalized intensities of trichoplein/GAPDH are shown as mean from three independent biological replicates. Percentages of ciliated cells and cyclin A-positive cells are shown as mean ± SD from three independent biological replicates (*n* > 200 each). **d** Control or USP8 (#1 or #2) knockdown RPE1 cells were treated with 200 nM cycloheximide-supplemented normal medium (10% FBS) for indicated times. Normalized intensities of trichoplein/GAPDH (mean ± SD from three independent technical replicates) were evaluated by immunoblotting analysis. To accurately evaluate the kinetics of trichoplein turnover, the samples were re-evaluated in Supplementary Fig. 4a. **p < 0.01, *0.01 < *p* < 0.05, n.s. not significant, two-tailed unpaired student’s *t*-tests

To identify DUBs that negatively regulate ciliogenesis in growing cells, we performed the following screens using hTERT-immortalized human retinal epithelia (RPE1) cells (see flowchart in Fig. 1a). In the primary screen, we used a Human ON-TARGETplus siRNA library^TM^ that consists of 86 pools of four siRNAs targeting each DUB. In the presence of serum, ciliogenesis was rarely observed in control cells, but significantly induced when one of the six genes encoding, *UCHL3, USP8, USP38, USP43, USP52*, and *USP54*, was downregulated (Supplementary Fig. 1a). To minimize false positives, we conducted the secondary screen using two individual siRNAs per the six DUBs, and obtained similar results (Supplementary Fig. 1b).

In the third screen, we tested if overexpression of these DUBs could suppress the serum starvation-induced ciliogenesis in RPE1 cells (Supplementary Fig. 2). FLAG-tagged UCHL3, USP8, USP38, and USP43 significantly blocked the ciliogenesis in their DUB activities-dependent manner. FLAG-USP52 and -USP54, both of which are assumed to have no DUB activity^30^, also prevented the ciliogenesis. From these results, we conclude that the six DUBs are negative regulators of ciliogenesis.

### USP8 depletion decreases the protein level of trichoplein

Among the six DUBs, we focused on USP8 because its knockdown not only induced unscheduled ciliogenesis but also decreased the protein level of trichoplein (Fig. 1b, c). The USP8-depleted cells also resulted in cell cycle arrest, as judged by the marked reduction of cyclin A-positive cells (Fig. 1b, c; bottom). The results seem reasonable since unscheduled ciliogenesis causes cell cycle arrest in RPE1 cells^13,16,25,26^, while also raising a concern that the observed loss of trichoplein and ciliogenesis may be merely consequences of cell cycle arrest. To address this issue, we examined the effects of USP8 knockdown in non-ciliated cells. Technically, we transfected with siRNA targeting IFT20 (intraflagellar transport protein 20) or Cep164, both of which are essential factors for ciliogenesis^21,25,31–33^, for 24 h prior to transfection with USP8 siRNA in RPE1 cells. Depletion of IFT20 or Cep164 almost completely abrogated the ciliogenesis, and rescued the cell cycle arrest, but the trichoplein levels remained low (Fig. 1b, c). These data clearly indicate that USP8 depletion induces the loss of trichoplein and causes the ciliogenesis-dependent cell cycle arrest.

Knockdown of the other five DUBs (UCHL3, USP38, USP43, USP52, and USP54) also decreased trichoplein levels, but these effects were reverted by IFT20 co-depletion (Supplementary Fig. 3). Thus, the loss of trichoplein appears to arise as a secondary effect of ciliogenesis. In other words, the five DUBs are unlikely to directly control trichoplein levels.

### USP8 requires its DUB activity to block ciliogenesis

Treatment with cycloheximide gradually reduced the protein levels of trichoplein through its degradation^26^, and this rate was significantly accelerated by USP8 depletion (Fig. 1d and Supplementary Fig. 4a). By contrast, there was a marginal difference in the trichoplein messenger RNA levels between control and USP8-depleted cells (Supplementary Fig. 4b). These results suggest that USP8 knockdown facilitates degradation of trichoplein.

**Fig. 2:**
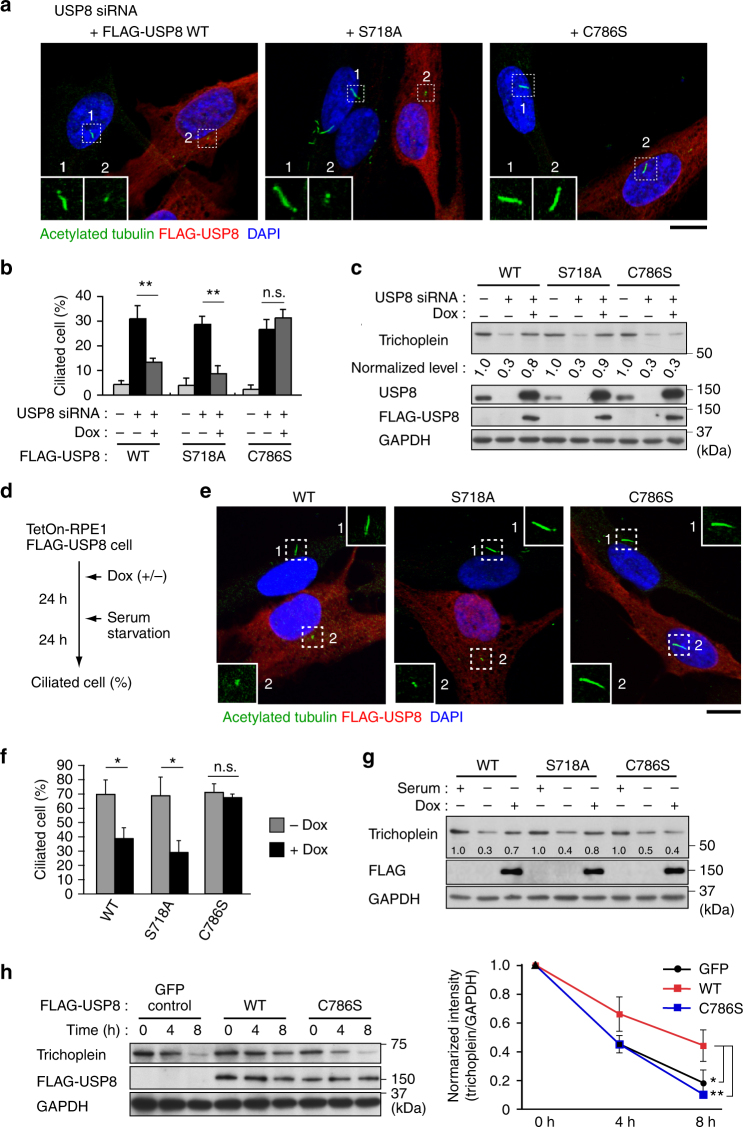
USP8 activity is essential for suppression of ciliogenesis and stabilization of trichoplein protein. **a–c** TetOn-RPE1 FLAG-USP8 cell lines (WT, S718A, and C786S) transfected with control or USP8 siRNA (#1) were cultured for 48 h in the presence or absence of doxycycline (Dox, 100 ng ml^−1^). **d–g** Expression of FLAG-USP8 (WT, S718A, and C786S) was induced by treatment with Dox (100 ng ml^−1^), and then subjected to serum starvation as shown in **d**. Representative confocal images of anti-acetylated-tubulin (green), anti-FLAG (red), and DAPI (blue) are shown in **a** and **e**. Insets are magnified images of dashed squares. Scale bars, 10 μm. Percentages of ciliated cells are shown as mean ± SD from three independent biological replicates (*n* > 200 each) in **b** and **f**. Immunoblotting analysis of trichoplein, USP8, FLAG and GAPDH are shown in **c** and **g**. Normalized intensities of trichoplein/GAPDH are shown as mean from three independent biological replicates. **h** FLAG-USP8 (WT or C786S) or GFP (as a control) were expressed by treatment with Dox (100 ng ml^−1^) in TetOn-RPE1 cells, and the cells were treated with 200 nM cycloheximde-supplemented serum-starved medium for indicated times. Normalized intensities of trichoplein/GAPDH are shown as mean ± SD from three independent technical replicates. **p < 0.01, *0.01 < *p* < 0.05, n.s. not significant, two-tailed unpaired student’s *t*-tests

To ask whether the USP8 knockdown phenotypes, such as the accelerated trichoplein degradation and ciliogenesis, depended upon the DUB activity, we established RPE1 cell lines that expressed siRNA-resistant FLAG-USP8 constructs in a doxycycline (Dox)-dependent manner. Expression of FLAG-USP8 wild-type (WT) and S718A, a catalytically active mutant that does not undergo 14-3-3-mediated catalytic inhibition^34^, effectively reverted both phenotypes of USP8-depleted cells, but C786S, a catalytically inactive mutant, had no effect (Fig. 2a–c). We further confirmed that overexpression of FLAG-USP8 blocked the serum starvation-induced trichoplein degradation and ciliogenesis in a DUB activity-dependent manner (Fig. 2d–h). Thus, USP8 requires its DUB activity to stabilize trichoplein and to suppress ciliogenesis.

Contrary to our data, Troilo et al.^35^ have reported that USP8 is a positive regulator of ciliogenesis. They demonstrated with RPE1 cells that USP8 knockdown prevented serum starvation-induced ciliogenesis, and proposed that this is due to the destabilization of HIF1α. We, therefore, scrutinized the effects of USP8 knockdown using additional four siRNAs. However, all the six siRNAs, some of which almost completely depleted USP8 from RPE1 cells, did not disturb serum starvation-induced ciliogenesis, and had the capability to induce unscheduled ciliogenesis in growth condition (Supplementary Fig. 5a–c). Similar results were also obtained with IMR90 human fibroblasts (Supplementary Fig. 5d–f). Moreover, we detected no reduction of HIF1α levels in USP8-depleted RPE1 cells (Supplementary Fig. 5a).

### USP8 knockout zebrafish develops in ciliopathy phenotypes

**Fig. 3:**
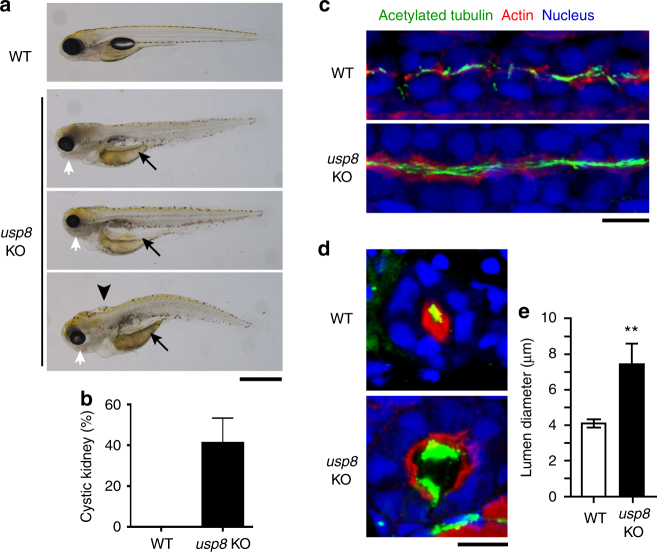
*usp8* knockout zebrafish displays ciliopathy-related phenotypes. **a** Wild-type (WT) and *usp8*-knockout (*usp8*-KO) zebrafish with cystic kidney (arrows), hydrocephalus (arrowhead), and microphthalmia (white arrows) at 5 dpf. Scale bar, 500 μm. **b** Incidence of cystic kidney in WT (*n* = 75) and *usp8* KO (*n* = 101) at 4 or 5 dpf. **c–e** Immunofluorescence analysis of cilia of pronephric duct of WT and *usp8*-KO at 27 hpf (**c**, sagittal plane) and at 4 dpf (**d**, transverse plane). Green, acetylated-tubulin; red, actin; blue: nuclei. Scale bars, 10 μm. Pronephric duct lumen diameter of WT (*n* = 11) and *usp8* KO (*n* = 7) at 4 dpf are shown as mean ± SD in **e**. **p < 0.01, two-tailed unpaired student’s *t*-tests

**Fig. 4:**
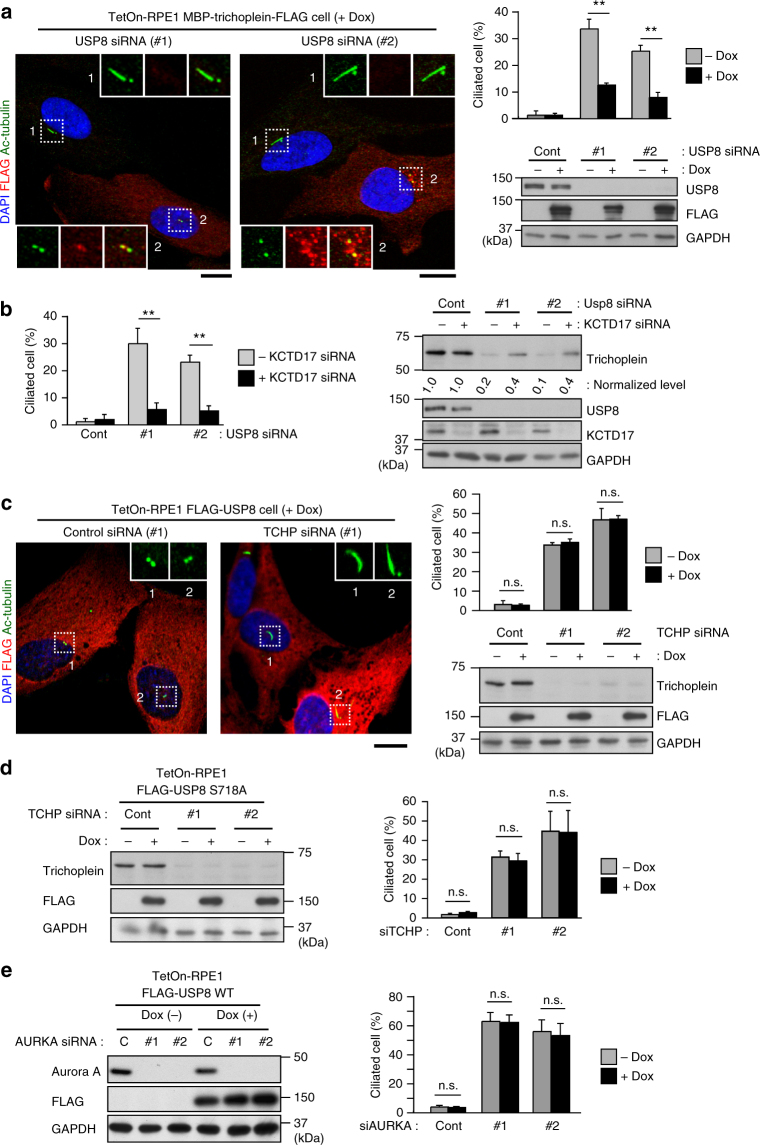
USP8 suppresses ciliogenesis by counteracting trichoplein degradation. **a** TetOn-RPE1 MBP-trichoplein-FLAG cells transfected with control or USP8 siRNA (#1 or #2) were cultured for 48 h in the presence or absence of doxycycline (Dox, 30 ng ml^−1^). **b** Sixteen hours after transfection with or without KCTD17 siRNA, RPE1 cells were further transfected with control or USP8 siRNA (#1 or #2), and then cultured for 32 h. **c–e** TetOn-RPE1 FLAG-USP8 WT (**c**,** e**) or S718A (**d**) cells were transfected with trichoplein (**c**,** d**; siTCHP, #1 or #2) or Aurora A siRNA (**e**; siAURKA). Six hours after transfection, these cells were treated with 100 ng ml^−1^ of Dox. Representative confocal images of acetylated-tubulin (green), FLAG (red) and DAPI (blue), percentages of ciliated cells, and immunoblotting analysis are shown. Normalized intensities of trichoplein/GAPDH are shown as mean from three independent biological replicates in **b**. Graphs represent mean ± SD from three independent biological replicates (*n* > 200 each). **p < 0.01, *0.01 < *p* < 0.05, n.s. not significant, two-tailed unpaired student’s *t*-tests. Scale bars, 10 μm

Next, we aimed to assess whether USP8 is involved in ciliary regulation in vivo and in ciliopathy conditions. We evaluated this in zebrafish, an excellent model organism for the study of cilia structure and function. Using CRISPR/Cas9, we generated *usp8* knockout (KO) zebrafish (Supplementary Fig. 6), which displayed various ciliopathy-related phenotypes, including cystic kidney, hydrocephalus, and microphthalmia (Fig. 3a). The most frequent ciliopathy-related phenotype observed in *usp8* KO was cystic kidney (Fig. 3b). Immunohistochemical staining revealed the dilation of pronephric duct at 27 h post-fertilization (hpf) (Fig. 3c) and 4 days post-fertilization (dpf) (Fig. 3d, e) compared with WT zebrafish. The length of pronephric cilia in usp8 KO zebrafish seems to be longer than that of WT zebrafish at 27 hpf (Fig. 3c) and 4 dpf (Fig. 3d). These in vivo studies support the in vitro finding that USP8 functions to suppress ciliogenesis and suggest that malfunction of USP8 cause ciliopathy through elongation of cilia. Taken together, USP8 functions as a negative, but not a positive, regulator of ciliogenesis.

### USP8 suppresses ciliogenesis by stabilizing trichoplein

Then, we assessed whether the USP8-regulated ciliogenesis depended upon the trichoplein levels. Dox-induced expression of MBP-trichoplein-FLAG significantly prevented ciliogenesis in USP8-depleted cells (Fig. 4a), but the subsequent removal of Dox allowed the cells to form cilia in response to the trichoplein degradation (Supplementary Fig. 7). Moreover, USP8 depletion-induced ciliogenesis was reverted by depletion of KCTD17, a subunit of the E3 ligase that ubiquitinates trichoplein^23,26^, blocking the degradation of trichoplein (Fig. 4b). Inversely, overexpression of FLAG-USP8 did not rescue ciliogenesis caused by knockdown of trichoplein or Aurora A (Fig. 4c–e). These observations indicate that USP8 acts upstream of the trichoplein-Aurora A pathway to suppress ciliogenesis.

**Fig. 5:**
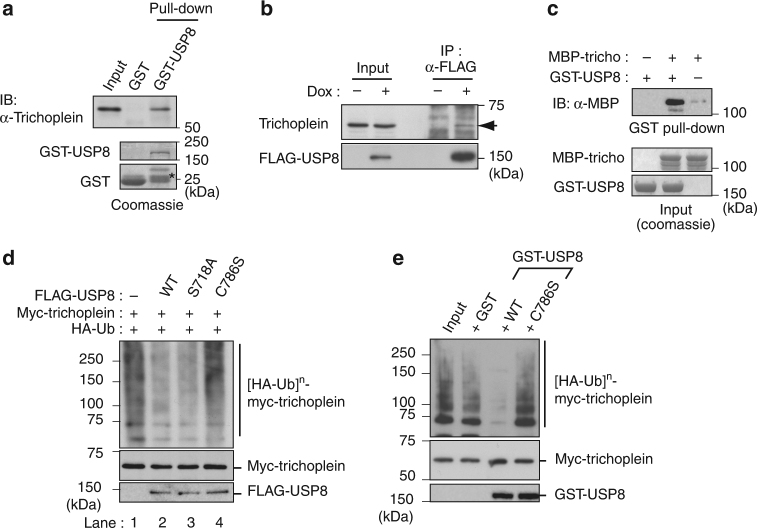
USP8 binds and deubiquitinates trichoplein. **a** Bacterially purified GST or GST-USP8 were incubated with RPE1 cell lysates, and then affinity purified with glutathione-sepharose. The samples were analyzed by immunoblotting with anti-trichoplein and by coomassie staining. Asterisk indicates fragmented GST-USP8. **b** Endogenous trichoplein (indicated by arrow) was co-immunoprecipitated with anti-FLAG antibody form TetOn-RPE1 FLAG-USP8 WT cells that were treated with, but not without, doxycycline (Dox: 100 ng ml^−1^). **c** GST-USP8 was incubated with MBP-trichoplein in vitro, and then affinity purified with glutathione-sepharose. **d** Myc-trichoplein and HA-ubiquitin were co-transfected with or without FLAG-USP8 (WT, S718A, or C786S) in HEK293T cells. Six hours after treatment with 10 μM MG132, anti-Myc immunoprecipitates were analyzed by immunoblotting with anti-HA and anti-Myc. Amounts of FLAG-USP8 proteins were analyzed in cell lysates. **e** Polyubiquitinated Myc-trichoplein ([HA-Ub]^n^-Myc-trichoplein) was immunoprecipitated from HEK293T cells transfected with Myc-trichoplein and HA-ubiquitin (input), as described in **d**, and then incubated with GST or GST-USP8 (WT or C786S) for 1 h at 37 °C

We, therefore, tested if USP8 bound trichoplein. Pull-down assays showed that bacterially purified GST-USP8 bound trichoplein in RPE1 lysates (Fig. 5a). Co-immunoprecipitation assays also demonstrated binding between trichoplein and FLAG-USP8 (Fig. 5b). Interaction between the two proteins was direct, as recombinant MBP-trichoplein was co-affinity purified with GST-USP8 in vitro (Fig. 5c). We further narrowed down their binding regions: a carboxyl-terminal USP8 fragment (714-1,118), which mainly contains the catalytic domain, bound an amino-terminal trichoplein fragment (1–38) that is close to its ubiquitination sites, Lys-50 and Lys-57^26^ (Supplementary Fig. 8). Consistent with their binding, expression of FLAG-USP8 (714-1,118) rescued the trichoplein degradation and the ciliogenesis of USP8-depleted cells (Supplementary Fig. 9).

We addressed whether trichoplein was a substrate of USP8. Myc-trichoplein was strikingly polyubiquitinated by co-expression of HA-ubiquitin in HEK293T cells^26^ (Fig. 5d; lane 1), but the ubiquitination levels were obviously decreased when FLAG-USP8 WT or S718A, but not C786S, was further co-expressed (Fig. 5d; lanes 2–4). We next immunopurified the polyubiquitinated myc-trichoplein, and incubated it with purified GST-USP8 proteins in vitro (Fig. 5e). Incubation with the WT protein resulted in almost complete deubiquitination of myc-trichoplein, whereas the C786S mutant had no effect. In conclusion, USP8 directly binds and deubiquitinates trichoplein.

### The USP8 activity is decreased upon serum starvation

**Fig. 6:**
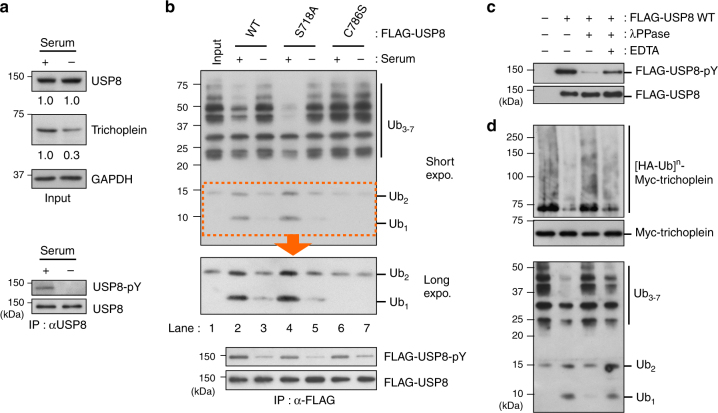
Serum starvation induces dephosphorylation and inactivation of USP8. **a** USP8 was immunoprecipitated from PRE1 cells cultured in serum-fed medium (serum: + ) or subjected to 24 h serum starvation (serum: -), and the phospho-tyrosine (pY) levels of anti-USP8 immunoprecipitates were analyzed by immunoblotting. The levels of trichoplein, USP8, GAPDH in cell lysates (input) were also analyzed, and normalized intensities of trichoplein/GAPDH and USP8/GAPDH are shown as mean from three independent biological replicates. **b** FLAG-USP8 variants (WT, S718A, and C786S) were immunopurified from serum-fed (+) or -starved (-) TetOn-RPE1 cells, and then incubated with ubiquitin oligomers (Ub_3–7_) for 2 h at 37 °C. Anti-ubiquitin, anti-phospho-tyrosine (pY), and anti-FLAG immunoblotting are shown. **c** FLAG-USP8 WT immunopurified from serum-fed RPE1 cells (lane 2), were treated with λPPase in the absence (lane 3) or presence (lane 4) of EDTA, and analyzed by anti-pY and anti-FLAG immunoblotting. **d** FLAG-USP8 WT proteins prepared in **c** were incubated with ubiquitin oligomers (Ub_3–7_; top) or polyubiquitinated myc-trichoplein ([HA-Ub]^n^-myc-trichoplein; bottom) for 2 h at 37 °C. Amounts of myc-trichoplein in the reactions are shown in a middle panel

It remains unclear how serum starvation terminates the USP8-mediated stabilization of trichoplein. USP8 expression levels were reported to become undetectable by serum starvation in WI-38 human fibroblasts^36^, however, we found no change in RPE1 cells (Fig. 6a; upper panels). We, therefore, examined whether serum starvation affected the USP8 activity in RPE1 cells. Equal amounts of FLAG-USP8 variants were immunoprecipitated from serum-fed or -starved cells, and then incubated with Lys48-linked ubiquitin oligomers (Ub_3–7_) in vitro. Immunoprecipitation of the WT protein from serum-fed cells cleaved ubiquitin oligomers and yielded dimmers (Ub_2_) and monomers (Ub_1_) (Fig. 6b; compare lane 1 with 2). However, when immunoprecipitated from serum-starved cells, the activity was significantly attenuated (Fig. 6b; lane 3). C786S mutant had no effect on the cleavage of ubiquitin oligomers, regardless of the culture conditions (Fig. 6b; lanes 6 and 7). These results suggest that USP8 is enzymatically inactivated upon serum starvation. Although the USP8 activity is known to be inhibited through Ser-718 phosphorylation-mediated 14-3-3 binding^34^, this inhibitory mechanism was not likely to function at least during ciliogenesis, because S718A activity was also reduced by serum starvation (Fig. 6b; lanes 4 and 5).

Of note, serum starvation decreased the Tyr-phosphorylation levels of USP8 (Fig. 6a; lower panels) and FLAG-USP8 proteins (Fig. 6b; lower panels). We, therefore, assessed whether this influenced USP8 activity. FLAG-USP8 WT protein was immunopurified from serum-fed RPE1 cells, and then a fraction of the protein was treated with λ protein phosphatase (λPPase) to prepare a dephosphorylated variant (Fig. 6c). FLAG-USP8 containing Tyr-phosphorylated form efficiently deubiquitinated both the polyubiquitinated myc-trichoplein and ubiquitin oligomers in vitro, but showed the limited effect after dephosphorylation (Fig. 6d). These data suggest that USP8 phosphorylation, possibly on Tyr residue(s), enhances its DUB activity.

### EGFR activates USP8 by phosphorylating Tyr-717 and Tyr-810

**Fig. 7:**
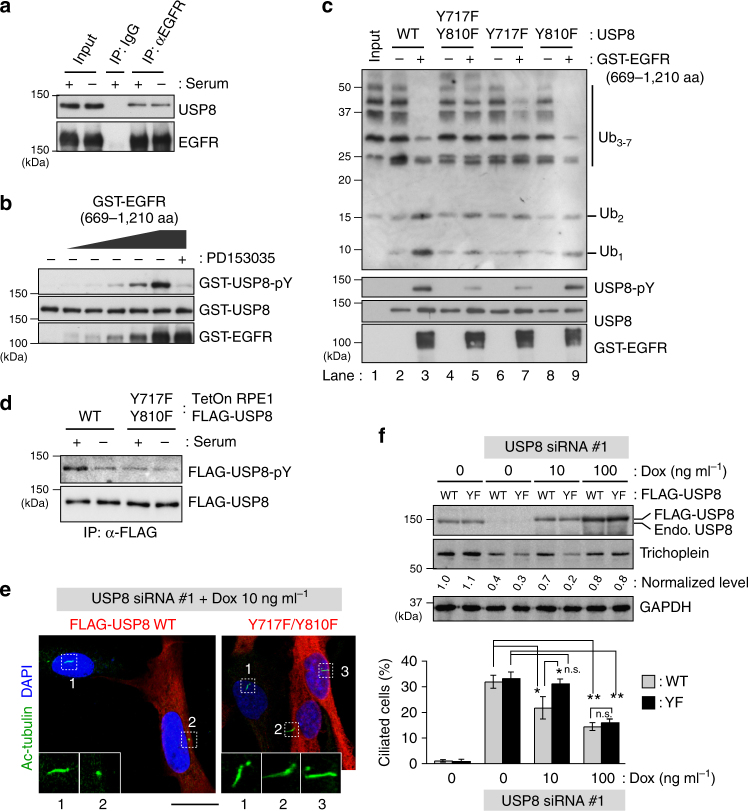
EGFR directly phosphorylates USP8 on Tyr-717 and Tyr-810 to elevate its DUB activity. **a** Anti-EGFR immunoprecipitates from serum-fed (serum: + ) or -starved (serum: -) RPE1 cell lysates were analyzed by anti-USP8 and anti-EGFR immunoblotting. **b** Bacterially purified GST-USP8 was incubated with purified GST-EGFR (669–1210 aa) in the presence or absence of 1 μM PD153035 for 15 min at 30 °C. **c** Bacterially purified non-tagged USP8 variants (WT, Y717F/Y810F, Y717F, and Y810F) were incubated with or without GST-EGFR (669–1210 aa) for 15 min at 30 °C, and then incubated with ubiquitin oligomers (Ub_3–7_) for 15 min at 37 °C. **d** TetOn-RPE1 FLAG-USP8 (WT and Y717F/Y810F) cells treated with 10 ng ml^−1^ of Dox (serum: + ) were subjected to 24 h serum starvation (serum: −). Anti-pY and anti-FLAG immunoblotting of anti-FLAG immunoprecipitates are shown. **e**,** f** TetOn-RPE1 FLAG-USP8 (WT and Y717F/Y810F; YF) cells were transfected with control or USP8 siRNA (#1), and then cultured for 48 h in the presence of indicated concentration of Dox. Representative confocal images of acetylated-tubulin (green), FLAG (red) and DAPI (blue) are shown in **e**. Scale bar, 20 μm. Normalized intensities of trichoplein/GAPDH are calculated by immunoblotting (**f**) and shown as mean from three independent biological replicates. Percentages of ciliated cell (mean ± SD from three independent experiments, *n* > 200 each) are shown in **f**. Normalized intensities of trichoplein/GAPDH are shown as mean from three independent biological replicates. **p < 0.01, *0.01 < *p* < 0.05, n.s., not significant, two-tailed unpaired student’s *t*-tests

To fully understand the underlying mechanism of USP8 activation, we searched for a Tyr kinase that is responsible for the phosphorylation of USP8. Here, we focused on epidermal growth factor receptor (EGFR) kinase because it has been reported to bind and phosphorylate USP8^34,37–39^. Indeed, USP8 co-immunoprecipitated with EGFR from RPE1 cell lysates (Fig. 7a). Furthermore, recombinant GST-EGFR (cytosolic region; 669–1210) directly phosphorylated GST-USP8 in the absence of an EGFR-specific inhibitor, PD153035 (Fig. 7b). To validate the effect of EGFR phosphorylation of USP8 in vitro, we used also non-tagged USP8 because GST-USP8 had a significantly higher DUB activity than non-tagged USP8 in non-phosphorylated forms (Supplementary Fig. 10). Importantly, GST-EGFR phosphorylated non-tagged USP8 and elevated its DUB activity toward ubiquitin oligomers (Fig. 7c; lanes 1–3), but GST-EGFR did not activate GST-USP8 (Supplementary Fig. 10). The fused GST might perturb the regulatory mechanism of USP8. An alternative interpretation would be that it is already active once dimerized via GST. In vitro phosphorylation assays identified Tyr-717 and Tyr-810 as the major phosphorylation sites of USP8 (Supplementary Fig. 11). Substitution of the two Tyr residues to Phe (Y717F/Y810F) reduced the phosphorylation levels of FLAG-USP8 in growing RPE1 cells (Fig. 7d). We, therefore, examined the effect of Y717F/Y810F mutation on DUB activity. Bacterially purified non-tagged USP8 WT and Y717F/Y810F proteins exhibited similarly low DUB activities (Fig. 7c; lanes 2 and 4). Treatment with GST-EGFR elicited the phosphorylation and activation of the WT, while the Y717F/Y810F activity was only marginally affected in accordance with its low level of Tyr-phosphorylation (Fig. 7c; lanes 3 and 5). A single mutation at either site had modest or weak effects (Fig. 7c; lanes 6–9). Taken together, EGFR activates USP8 by directly phosphorylating Tyr-717 and −810 in vitro. We further observed that other receptor tyrosine kinases (RTKs), such as PDGFRα, PDGFRβ and FGFR1, were also capable for inducing the phosphorylation-mediated USP8 activation in vitro (Supplementary Fig. 12).

### EGFR-USP8-trichoplein signaling suppresses ciliogenesis

We then analyzed the roles of EGFR-mediated phosphorylation of USP8 in ciliogenesis. As described earlier, USP8 knockdown induced trichoplein degradation and unscheduled ciliogenesis, whereas these phenomena were ameliorated by expression of endogenous level of FLAG-USP8 WT (Fig. 7e, f; 10 ng ml^−1^ of Dox). In contrast, equal levels of Y717F/Y810F had only a modest effect, suggesting that phosphorylations of Tyr-717 and Tyr-810 are important for trichoplein-mediated inhibition of ciliogenesis. Because Y717F/Y810F retains week activity (Fig. 7c), its overexpression could rescue the knockdown phenotypes (Fig. 7f; 100 ng ml^−1^ of Dox).

**Fig. 8:**
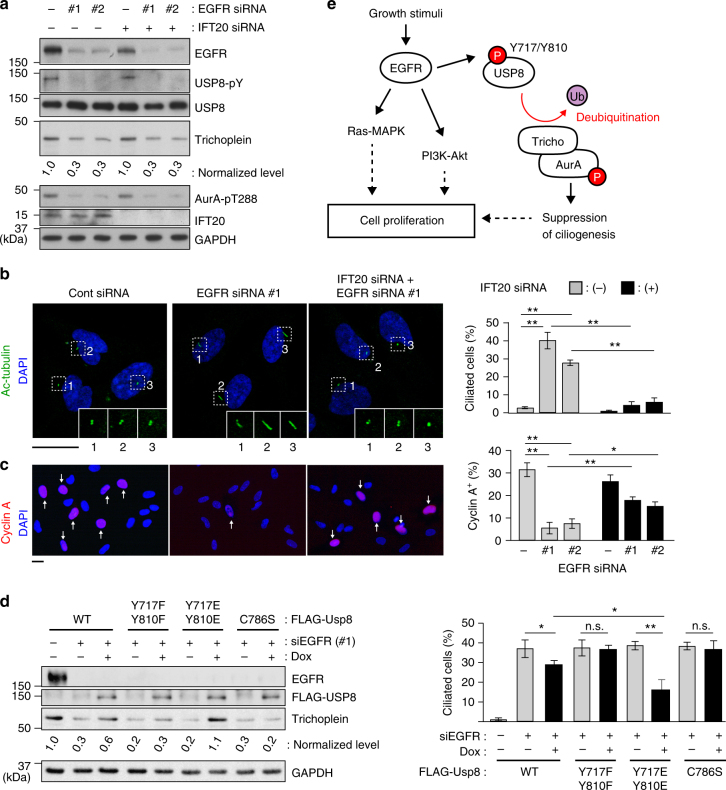
EGFR contributes cell cycle progression through USP8-trihocplein pathway-mediated cilia suppression. **a–c** Twenty-four hours after transfection with control or IFT20 siRNA, RPE1 cells were further transfected with control or EGFR siRNA (#1 or #2) and then cultured for 48 h in normal medium (10% FBS). The cells were analyzed by immunoblotting with indicated antibodies (**a**), and immunofluorescence staining with anti-acetylated-tubulin and anti-cyclin A to evaluate percentages of ciliated cell (**b**) and cyclin A-positive cell (**c**), respectively. Scale bars, 20 μm. **d** TetOn-RPE1 FLAG-USP8 (WT, Y717F/Y810F, Y717E/Y810E, C786S) cells were transfected with control or EGFR siRNA (#1) and then cultured for 48 h in the presence or absence of Dox (10 ng ml^−1^). Immunoblotting analysis with indicated antibodies (left) and percentages of ciliated cell (right) are shown. **e** Proposed model: normalized intensities of trichoplein/GAPDH in **a** and **d** are shown as mean from three independent biological replicates. Graphs represent mean ± SD from three independent experiments (*n* > 200 each). **p < 0.01, *0.01** < ***p* < 0.05, n.s. not significant, two-tailed unpaired student’s *t*-tests

Consistent with the in vitro assays, EGFR knockdown significantly reduced the Tyr-phosphorylation levels of USP8 in serum-fed RPE1 cells (Fig. 8a). The levels of trichoplein and Aurora A kinase activity were also decreased, and the cells formed cilia and exited from cell cycle (Fig. 8a–c). We, therefore, determined whether the EGFR knockdown phenotypes were rescued by expression of endogenous levels of FLAG-USP8 variants (Fig. 8d). Expression of WT USP8 modestly rescued the trichoplein degradation and ciliogenesis probably because its DUB activity was declined without EGFR. As expected, Y717F/Y810F or C786S expression had no effect. Notably, expression of Y717E/Y810E, a phosphomimetic mutant, rescued effectively. Furthermore, expression of MBP-trichoplein-FLAG also blocked the unscheduled ciliogenesis of EGFR-depleted cells (Supplementary Fig. 13). Thus, EGFR knockdown induces ciliogenesis by downregulating the USP8-trichoplein pathway.

Importantly, the cell cycle arrest of EGFR knockdown cell was alleviated when ciliogenesis was abrogated by depletion of IFT20 or Cep164 (Fig. 8a–c and Supplementary Fig. 14), indicating that the EGFR-mediated cell proliferation is coordinated not only by the well-known kinase cascades, such as the Ras/MAPK or PI3K/Akt pathways^40,41^, but also by suppression of ciliogenesis (Fig. 8e).

### The EGFR signal in serum-induced ciliary disassembly

Finally, we were wondering if the EGFR signal would participate in serum-induced cilia disassembly (Supplementary Fig. 15). Serum stimulation of serum-starved RPE1 cells leads to ciliary disassembly, which allows the cells to restart the cell cycle progression^18^. The cells also demonstrated the increased levels of USP8 phosphorylation and trichoplein expression, in accordance with the EGFR autophosphorylation. Treatment with EGF alone, which robustly up-regulated the EGFR-USP8-trichoplein pathway, also induced ciliary disassembly and cell cycle re-entry, although less efficient than serum treatment, in contrast to the previous report^18^. Meanwhile, treatment with lysophosphatidic acid, the major mitogen in serum, disassembled cilia sufficiently, despite with the relatively low, but significant, phosphorylation levels of EGFR and USP8. Thus, as described previously^13,17–23^, serum-induced ciliary disassembly is regulated by multiple signaling pathways, in which the EGF signal is likely to be involved.

## Discussion

The nature of growth-promoting signals that mediate suppression of ciliogenesis in dividing cells have remained largely unknown. In this study, we show that EGFR activation is responsible for this process by directly phosphorylating USP8 on Tyr-717 and Tyr-810. USP8 is activated by these phosphorylations and thereby deubiquitinates and stabilizes trichoplein. Thus, serum starvation triggers downregulation of the EGFR-USP8 signaling and then switches to CRL3^KCTD17^-mediated polyubiquitination and degradation of trichoplein, which leads to Aurora A inactivation, the decisive step for ciliogenesis^23,25,26^ (summarized in Fig. 8e). Moreover, *usp8* knockout zebrafish displays the ciliopathy-related phenotypes (Fig. 3). These data collectively indicate that the EGFR-USP8-trichoplein-Aurora A axis is one of the critical signaling cascades that regulate ciliogenesis.

Our siRNA-based functional screens have identified six DUBs, including USP8, USP38, USP43, USP52, USP54, and UCHL3, as negative regulator for ciliogenesis (Supplementary Figs. 1–3). Further studies reveal that among the six DUBs, USP8 is a DUB for trichoplein. In contrast, our data suggest that the other five DUBs control ciliogenesis independently of trichoplein since their knockdown has a negligible effect on trichoplein levels in non-ciliated cells (Supplementary Fig. 3). Recent studies have shown that a variety of ciliary proteins, such as Ndel1^23^, NDE1^42^, Nek1^43^, and DVL2^44^, are polyubiquitinated during ciliogenesis, and we confirmed that at least Ndel1 is not a substrate of USP8 (unpublished data). Given that USP38, USP43, and UCHL3, but not USP52 and USP54, require their DUB activities to suppress ciliogenesis, they might act on deubiquitination of these ciliary proteins.

USP8 is activated by EGFR-mediated phosphorylation at Tyr-717 and Tyr-810, and this phosphorylation is essential for suppressing ciliogenesis (Figs. 7 and 8). In RPE1 cells, EGFR knockdown is sufficient to repress the USP8-trichoplein-Aurora A axis and induce ciliogenesis, however, we do not exclude the involvement of other RTKs in other cell types since USP8 is also activated by PDGFRs and FGFR1-mediated phosphorylation in vitro (Supplementary Fig. 12).

Our conclusion is supported by the data of *usp8* KO zebrafish, in which cystic kidney occurs in ~40% of the KO animals (Fig. 3a, b). A unifying pathogenic concept suggests that all products of mutated genes causing cystic kidney are expressed in primary cilia and/or centrosome^45^. It has been reported that trichoplein is localized at the centrosome^25,28^ and that USP8 interacts with centrosome-associated proteins^46^. The relatively high incidence of cystic kidney in *usp8* KO zebrafish is well concordant with the unifying theory. Immunofluorescence analysis of *usp8* KO zebrafish also revealed that the cilia length and the lumen diameter of pronephric duct are longer and wider than those of WT zebrafish (Fig. 3c–e). While many ciliopathies result from shortening of cilia, abnormal elongation of cilia can also cause ciliopathy, although the functional consequences of cilia elongation are not clear^47,48^. For example, depletion of RPGRIP1L increase cilia length^49^. Defects of RPGRIP1L can cause of Joubert syndrome type 7 and Meckel syndrome type 5^50,51^. Cystic kidney, hydrocephalus, and microphthalmia, which were observed in usp8 KO zebrafish (Fig. 3a), are also accompanied in both Joubert and Meckel syndromes^52,53^. These findings suggest that defects of USP8 may cause ciliopathy through elongating primary cilia.

It is well-known that RTKs participate in cell proliferation through growth signaling transduction pathways, such as Ras/MAPK and PI3K/Akt. In this study, we demonstrate that primary cilia abrogation, which is caused by depletion of IFT20 or Cep164, ameliorates the cell cycle arrest of EGFR-depleted cells (Fig. 8a–c and Supplementary Fig. 14). Thus, EGFR contribute in a significant way to cell cycle progression by inhibiting ciliogenesis. Our findings have important implications for the causal relationship between excessive RTKs activities^40,41^ and defective ciliogenesis^8^, frequent characteristics of certain cancer cells, and provide a valuable insight into the molecular mechanisms of a reciprocal relationship between primary cilia and cell proliferation.

## Methods

### Antibodies

Commercial antibodies were as follows: For immunoblotting, mouse anti-cyclin A (1:1000, clone 25, BD Biosciences), anti-DYKDDDDK HRP-conjugate (1:4000, 1E6, WAKO), anti-EGFR (1:2000, 6F1, MBL), anti-FLAG (1:10,000 M2, Sigma-Aldrich), anti-GFP (1:2000, clones 7.1 and 13.1, Roche), anti-GST (1:1000, B-14, Santa Cruz Biotechnology), anti-HA-tag mAb-HRP-DirecT (1:4000, MBL), anti-MBP (1:1000, 1G12, MBL), anti-Myc peroxidase conjugated (1:2000, MC045, Nacalai tesque), anti-trichoplein (1:1000, G-2, Santa Cruz Biotechnology), anti-ubiquitin (1:1000, P4D1, Cell Signaling Technology), anti-UBPY/USP8 (1:1000, A-11, Santa Cruz Biotechnology), rabbit anti-trichoplein^25^ (1:2000), KCTD17^26^ (1:5000), USP8 (1:2000, D18F6, Cell Signaling Technology), anti-HIF1α (1:500, D2U3T, Cell Signaling Technology), anti-phospho-tyrosine (1:2000, p-Tyr-1000, Cell Signaling Technology), anti-IFT20 (1:500, ProteinTech), anti-phospho-Aurora A Thr-288 (1:500, C39D8, Cell Signaling Technology), anti-phospho EGFR Tyr-1068 (1:1000, D7A5, Cell Signaling Technology), anti-GAPDH (1:4000, 14C10, Cell Signaling Technology), and got anti-Cep164 (1:500, N-14, Santa Cruz Biotechnology) were used.

For immunoprecipitation assays, mouse anti-DYKDDDDK-tag antibody beads (1E6, WAKO), anti-EGFR (6F1, MBL), anti-Myc agarose conjugate (MC045, Nacalai tesque), rabbit anti-USP8, and normal mouse IgG control (Santa Cruz Biotechnology) were used.

For immunofluorescence, mouse anti-acetylated-α-tubulin (1:200, 6-11B-1, Sigma-Aldrich), anti-cyclin A (1:200, clone 25, BD Biosciences), FLAG (1: 2000, M2), rabbit anti-trichoplein^25^ (1:400) and γ-tubulin (1:500, Abcam) were used.

### Plasmids

Human cDNAs for USP8 (FLJ76940), Uchl3 (FLJ94247), Usp38 (FLJ44996), Usp43 (FLJ04332), Usp52 (FLJ86030), and Usp54 (FLJ04333) were obtained from the Human Gene and Protein Database (HGPD). For the transient expression of the above proteins, pDEST12.2 carrying each FLAG-tagged protein was constructed through the homologous recombination using GATEWAY technology (Invitrogen). Human cDNA for trichoplein was described previously^25^. pCGN-HA-ubiquitin was a kind gift from A. Kikuchi (Osaka University, Osaka, Japan). Transfection was performed with Lipofectamine 2000 (Invitrogen) or FuGENE HD (Promega) transfection reagents in HEK293T or RPE1 cells, respectively.

### Small-interfering RNAs (siRNAs)

Transfection of siRNA duplexes was performed with Lipofectamine RNAiMAX reagent according to the manufacturer’s protocol (Invitrogen). Each siRNA was used at final concentration of 40 nM (for IFT20) or 10 nM (for the others). Human ON-TARGETplus Deubiquitinating enzyme siRNA library SMARTpool (cat#: G-104705-025) and two individual siRNAs from corresponding SMARTpool reagents were purchased from Thermo Scientific. siRNAs targeting trichoplein, Aurora A, Cep164, EGFR, USP8, KCTD17, and IFT20 were from Qiagen. Target sequences are shown in Table S1.

### Cells

hTERT-RPE1 cells (originally derived from American Type Culture Collection (ATCC) clone CRL-4000) were grown at 37 °C in DMEM (Dulbecco's modified Eagle's medium) and F12 nutrient mix (1:1) supplemented with 10% fetal bovine serum (FBS, Hyclone) using 5% CO_2_ and 95% humidity. IMR-90 (ATCC CCL-186) and HEK293T (ATCC CRL-3216) were cultured in DMEM supplemented with 10% FBS.

TetOn RPE1 cell lines that expressed MBP-trichoplein-3xFLAG or FLAG-USP8 variants were established with the same procedure described previously^25^. The rtTA-advanced segment and the tTS transcriptional silencer segment from pTet-On advanced and pQC-tTS-IN (BD Biosciences Clontech) were recombined into the retroviral vector pDEST-PQCXIP and pDEST-PQCIN, respectively, by the LR reaction (Invitrogen) to generate PQCSIN-TetOn ADV and PQCXIP-tTS. The elongation factor 1 alpha promoter (EF) in CSII-EF-MCS (a gift from H. Miyoshi, RIKEN BioResource Center, Tsukuba, Japan) was replaced with a Tet-responsive promoter (TRE-Tight) from pTRE-Tight (BD Biosciences Clontech, San Jose, CA) followed by a modified RfA fragment (Invitrogen) to make a Tet-responsive lentivirus vector: CSII-TRE-Tight-RfA. Fusion cDNAs with siRNA-resistant trichoplein and USP8 were recombined into the lentiviral vector by the LR reaction to generate CSII-TRE-Tight-MBP-trichoplein-3xFLAG and CSII-TRE-Tight-FLAG-USP8, respectively. Doxycycline (Dox; Sigma-Aldrich) was added to induce the expression of MBP-trichoplein-3xFLAG and FLAG-USP8. Dox concentrations are indicated in the figure legends.

We confirmed that all these cell lines were not infected with mycoplasma.

### Reverse transcription-PCR (RT-PCR)

Semi-quantitative RT-PCR was performed using RNeasy Mini Kit (Qiagen,) and PrimeScript^TM^ RT reagent Kit (TaKaRa BIO), followed by PCR with human trichoplein-specific primers (QuantiTect Primer Assay, QT00067970, Qiagen) and GAPDH-specific primers (5′-GGCATGGCCTTCCGTGTTCCT-3′ and 5′-TCCTTGCTGGGGTGGGTGGTC-3′).

### Protein purification

FLAG-USP8 proteins expressed in TetOn RPE1 cells were immunoprecipitated with anti-DYKDDDDK-tag antibody beads. The immobilized FLAG-USP8 was treated with or without lambda protein phosphatase (λPPase) according to the manufacturer’s protocol (New England Biolabs), and then eluted with 100 μg ml^−1^ of FLAG peptide (Sigma-Aldrich) dissolved in PBS containing 0.1% Tween 20.

For purification from bacteria, USP8 cDNA constructs were inserted in frame to GST (glutathione S transferase) via the *Bam*HI/*Xho*I cloning site of pGEX-6P-3 (GE Healthcare). GST-USP8 proteins were expressed in in BL21 (DE3) *E. coli* stain (Stratagene) by 1 mM isopropyl β-d-1-thiogalactopyranoside induction at 30 °C overnight. MBP-trichoplein were expressed in BL21 CodonPlus RP strain (Agilent Technologies)^26^. Fusion proteins were purified through the affinity chromatography with glutathione-sepharose 4B (GE Healthcare) or amylose resin (New England Biolabs). For purification of non-tagged USP8, GST was removed from GST-USP8 by PreScission protease (GE Healthcare). GST-EGFR (669-1,210 aa), GST-PDGFRα (550-1,089 aa), GST-PDGFRβ (557-1,106 aa) and GST-FGFR1 (398-822 aa) were from Carna Bioscience.

### In vitro-binding assay

MBP-trichoplein (1 μg) and GST-USP8 (1 μg) were incubated in cell lysis buffer (20 mM Tris-HCl, pH 7.5, 150 mM NaCl, 2 mM β-glycerophosphate, 2 mM EGTA, and 1% Triton X-100) for 2 h at 4 °C, and then affinity purified with glutathione-sepharose 4B beads. After washing with cell lysis buffer three times, the beads were subjected to immunoblotting.

### GST pull-down assay

RPE1 cells were lysed in cell lysis buffer containing protease inhibitor cocktail (Nacalai Tesque), and then incubated with GST-USP8 (10 μg) or GST-14-3-3 (10 μg) for 2 h at 4 °C. The mixtures were subjected to affinity purification with glutathione-sepharose 4B beads. After washing with cell lysis buffer three times, the beads were subjected to immunoblotting.

### In vivo phosphorylation assay

One-hundred nanograms of GST-tagged receptor tyrosine kinases (RTKs) were incubated with 1 μg of bacterially purified GST-USP8 or non-tagged USP8 in 20 μl reaction buffer (25 mM Tris-HCl pH 7.5, 50 mM NaCl, 5 mM MgCl_2_, 1 mM MnCl_2_,10 μM ATP, 2 mM DTT) in the presence or absence of 0.1 mM [γ-^32^P] ATP at 30 °C for 10 min.

### In vivo ubiquitination assay

One day after transfection with indicated cDNA, cells were treated with 10 μM MG132 for 6 h to prevent the proteasomal degradation of polyubiquitinated trichoplein. The cells were lysed in the denaturing condition with hot ubiquitin buffer (95 °C) containing 25 mM Tris-HCl (pH 8.0), 1.5% SDS, 0.15% sodium deoxycholate, 0.15% NP-40, 1 mM EDTA, 1 μM okadaic acid and 5 mM N-ethylmaleimide. The lysates diluted (1:10) with cell lysis buffer were subjected to immunoprecipitation using agarose beads conjugated anti-Myc (MC045; Nacalai tesque, Japan). Immunoprecipitates were washed three times with Cell lysis buffer containing 0.1% SDS, and then analyzed by sodium dodecyl sulfate polyacrylamide gel electrophoresis (SDS-PAGE).

### In vitro deubiquitination assay of trichoplein

For preparation of polyubiquitinated trichoplein ([HA-Ub]^n^-myc-trichoplein), HEK293T cells transfected with myc-trichoplein and HA-ubiquitin were treated with 10 μM MG132 for 6 h, and then subjected to immunoprecipitation using anti-Myc (MC045) agarose conjugate (Nacalai, Japan) according to the “in vitro ubiquitination assay” section. The agarose beads were washed with deubiquitination buffer (20 mM Tris-HCl pH 7.5, 5 mM MgCl_2_, 1 mM MnCl_2_ and 2 mM DTT), and incubated in 20 μl of deubiquitination buffer containing 1.0 μg of GST-USP8 or 100 ng of FLAG-USP8 at 37 °C for indicated times in the figure legends.

### In vitro deubiquitination assay of ubiquitin oligomers

Non-tagged USP8 (1.0 μg), GST-USP8 (1.0 μg), or FLAG-USP8 (100 ng), prepared as described above, were incubated with 0.25 μg of Lys48-linked ubiquitin oligomers (Enzo Life Sciences) in 30 μl of deubiquitination buffer at 37 °C for indicated times in the figure legends.

### Immunoblotting and quantitation

For immunoblot analysis, cells were lysed in loading buffer (62.5 mM Tris-HCl pH 6.8, 2% SDS, 10% glycerol, 10% 2-mercaptethanol, and 0.01% bromophenol blue), separated by SDS-PAGE, transferred onto polyvinylidene difluoride (PVDF) membranes. Quantitative assessment of band intensity was performed using Image Lab statistical software (Bio-Rad) and ImageJ software (1.43r for Macintosh OS X; National Institute of Health). Uncropped versions of the most important blots are shown in Supplementary Fig. 16.

### Immunofluorescence cell staining

Immunofluorescence microscopy was performed using confocal microscopy (LSM510 META, Carl Zeiss) equipped with a microscope (Axiovert 200 M, Carl Zeiss), a plan Apochromat 100 × /1.4 NA oil-immersion lens, and LSM image browser software (Carl Zeiss), as described. In the most cases, cells cultured on sterile coverslips were fixed with −20 °C methanol for 10 min. For cyclin A staining, cells were fixed with 3.7% formaldehyde for 20 min at room temperature. For detection of primary cilia with anti-acetylated-tubulin antibody, cells were placed on ice for 30 min before methanol fixation.

### Knockout of usp8 in zebrafish

KO of usp8 in zebrafish was performed by the ready-to-use CRISPR/Cas9 method^54^. CRISPR RNAs (crRNAs) targeting usp8 genome, and trans-activating crRNA (tracrRNA) were obtained from FASMAC (Kanagawa, Japan). Recombinant Cas9 protein was obtained from Toolgen (Seoul, South Korea). In brief, crRNA, tracrRNA, and Cas9 protein were dissolved in sterilized water at concentrations of 250, 1000, and 1000 ng μl^−1^, respectively, and stored at −80 °C until required. For microinjection, the crRNA, tracrRNA, Cas9 protein, and a lissamine-labeled control morpholino with no known target gene (Gene Tools, Philomath, OR, USA) were mixed in Yamamoto’s Ringer’s solution (0.75% NaCl, 0.02% KCl, 0.02% CaCl2, 0.002% NaHCO3) to final concentrations of 100, 200, 400 ng μl^−1^, and 50 nM, respectively. The solution was injected into one- to four-cell-stage zebrafish embryos derived from the Tg (sox17:EGFP)^55^ or albino^56^ lines. Genomic DNA was extracted from the zebrafish by incubation in 50 μl of lysis buffer (10 mM Tris-HCl, pH 8.0, 0.1 mM EDTA, 0.2% Triton X-100, 200 μg ml^−1^ proteinase K) at 55 °C overnight, followed by incubation at 99 °C for 10 min. The solution was then placed at 4 °C and used as template for PCR. To detect the crRNA-induced mutations, we performed a heteroduplex mobility assay^57^. Briefly, a short fragment of the usp8 gene encompassing the crRNA target sites was amplified from the genomic DNA using primers and QuickTaq (Toyobo, Osaka, Japan). PCR cycling conditions were: 94 °C for 2 min followed by 40 cycles of 94 °C for 30 s, 60 °C for 30 s, and 68 °C for 30 s. The PCR products were electrophoresed on 10% polyacrylamide gels (Wako Chemicals) and visualized by ethidium bromide staining. The crRNA, tracrRNA, and PCR primer sequences are shown in Supplementary Table 2. Live larvae were photographed using a SMZ25 stereomicroscope (Nikon, Tokyo, Japan).

### Zebrafish analysis

Zebrafish at 27 hpf and 4 dpf zebrafish were fixed in a 4% paraformaldehyde/cacodylate-buffered solution (0.15 M, pH 7.4) containing 2 mM CaCl_2_ for overnight at 4 °C. The embryo or fishes samples were soaked in a cacodylate-buffered solution containing 2 mM CaCl_2_, 30% sucrose, and protease inhibitor cocktail tablets (Roche Diagnostics, Mannheim, Germany) at 4 ℃ for overnight. Then, the samples were quickly frozen in OCT compound (Sakura Finetek, Tokyo, Japan) and 12-μm thickness serial cryostat sections were cut and mounted on glass slides, then quickly blocked by 0.1 M phosphate buffer (pH 7.4) containing 4% Block Ace (DS Pharma Biomedical), protease cocktail, and 0.02% saponin, and incubated at room temperature (RT) for 20 min. Then, the samples were incubated at 4 ℃ for 1 overnight for primary Abs, and the 2 h at RT for secondary Abs. The Abs were diluted in 0.1 M phosphate buffer (pH 7.5) containing 0.02% saponin, 1% Block Ace, and protease cocktail. The dilutions of acetylated-tubulin and all the secondary Abs was 1:500. Images were captured by confocal laser scanning microscopy FV1000 system (Olympus) with 40 × and 60 × objective lenses and × 4 to × 7.0 zoom.

### Data availability

The data that support the findings of the current study are available from the corresponding author on reasonable request.

## Electronic supplementary material

Supplementary Information
