# Supplementary material for: EGF receptor kinase suppresses ciliogenesis through activation of USP8 deubiquitinase
Source: Nat Commun. 2018 Feb 22;9:758. doi: 10.1038/s41467-018-03117-y (PMC5823934; doi:10.1038/s41467-018-03117-y)
Supplement: Supplementary file 1 — Supplementary Information [file 41467_2018_3117_MOESM1_ESM.pdf]

Supplementary Information

EGF receptor kinase suppresses ciliogenesis through activation of USP8 deubiquitinase

Kasahara et al.

The Supplementary Information contains:

- ・Supplementary Figures 1-16
- ・Supplementary Tables 1 and 2

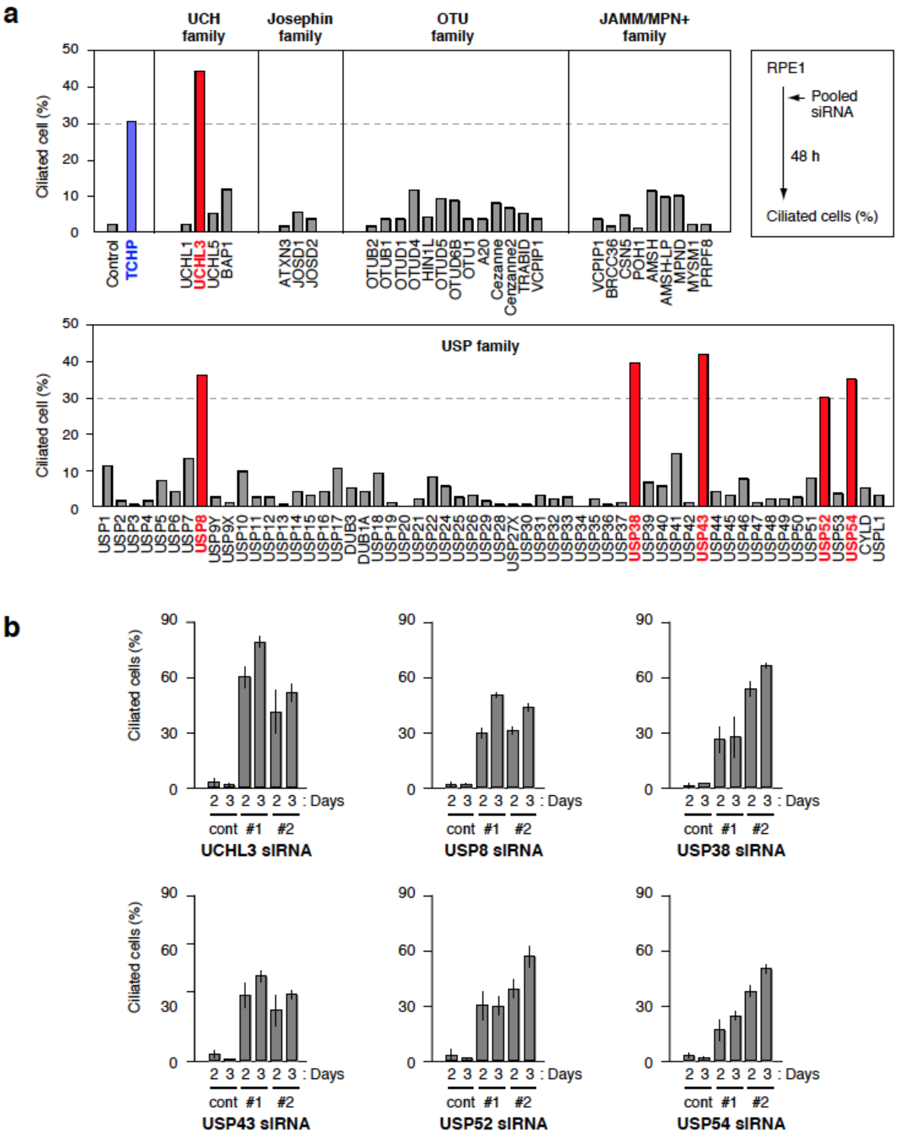

Supplementary Figure 1 | siRNA screens identify DUBs whose knockdown induces unscheduled ciliogenesis in RPE1 cells. (**a**) RPE1 cells were transfected with pooled siRNA for indicated DUBs, and then cultured for 48 h in normal medium (10% FBS). Percentages of ciliated cells are shown as mean from two or three independent biological replicates (n >200 each). Trichoplein knockdown (*TCHP*; a blue bar) serves as a positive control. The six DUBs whose knockdown significantly induces ciliogenesis are shown in red bars. (**b**) PRE1 cells were transfected with control (cont) or two-individual siRNAs (#1 and #2) for indicated DUBs, and then cultured for 48 or 72 h (2 or 3 days). Percentages of ciliated cells are shown as mean ± SD (from three to five independent biological replicates, n > 200 each).

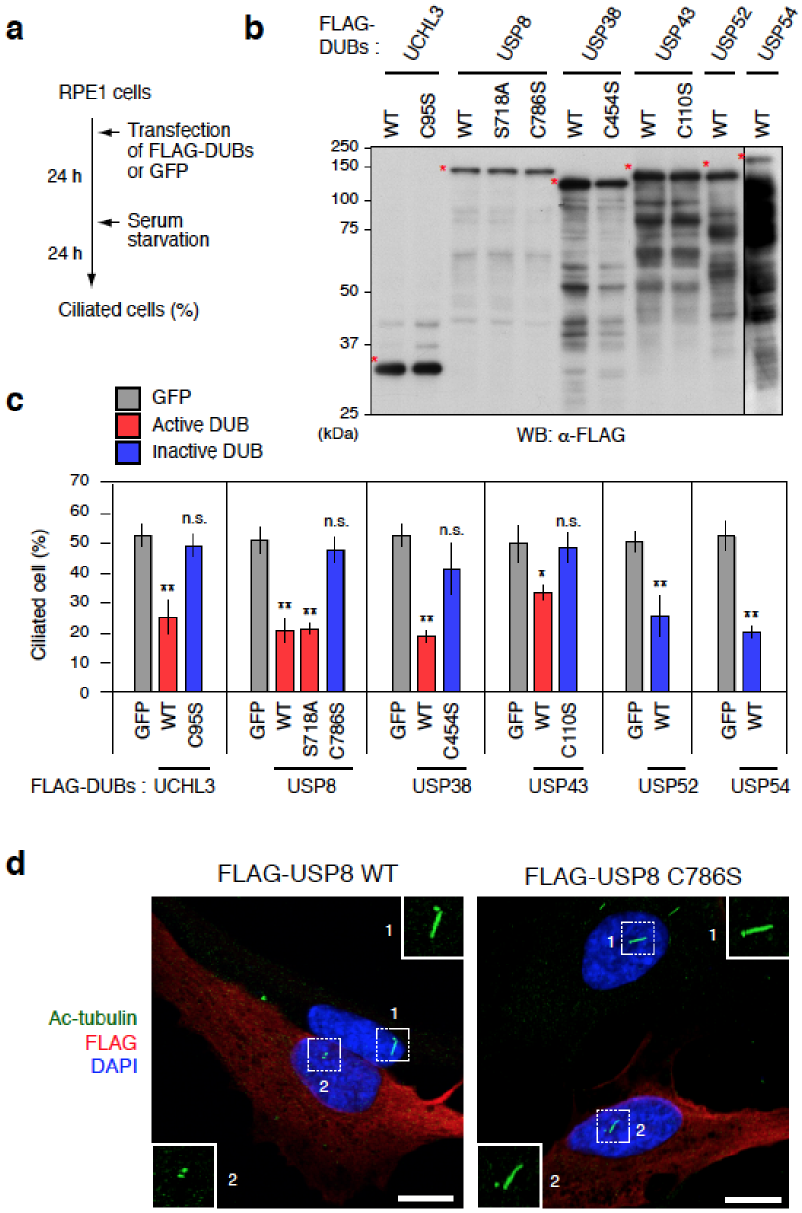

Supplementary Figure 2 | Overexpression of FLAG-tagged DUBs in serum-starved RPE1 cells. (**a**) Scheme of experimental procedure. (**b**) Expression of FLAG-DUBs, indicated by asterisks, were detected by anti-FLAG immunoblotting. (**c**) Percentages of ciliated cells are shown as mean ± SD (from three to five independent biological replicates, n > 200 each). **p<0.01, *0.01<P<0.05, n.s., not significant, two-tailed unpaired student's *t*-tests. (**d**) Representative confocal images of FLAG-USP8 (red), acetylated-tubulin (green) and DAPI (blue) are shown. Insets are magnified images of dashed squares. Scale bars, 10 μm.

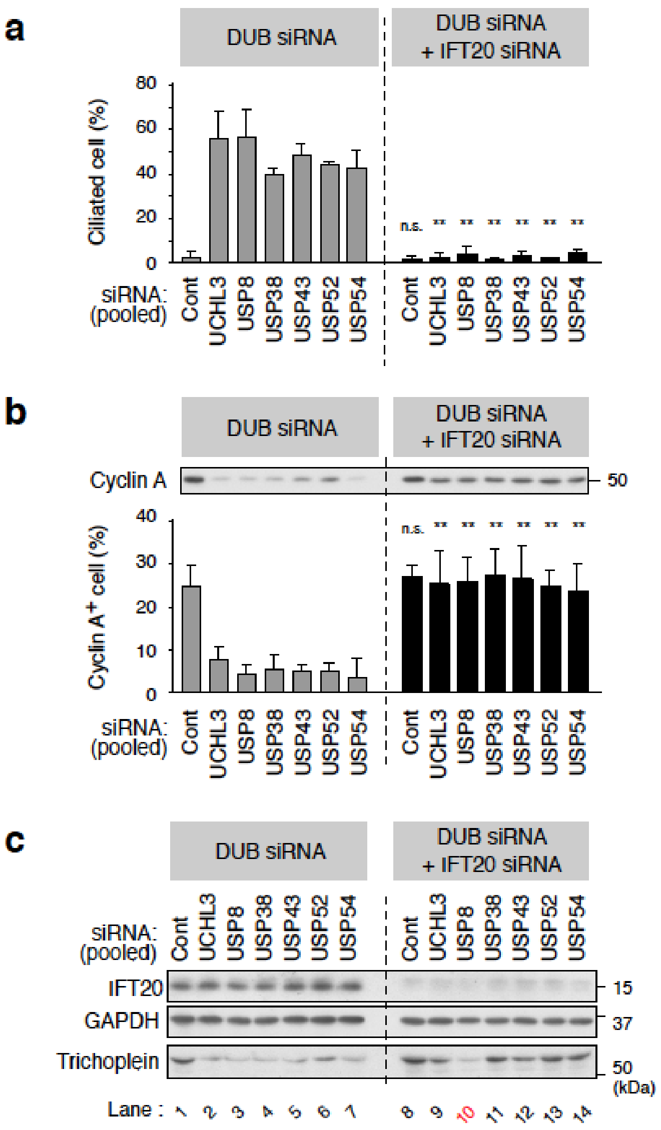

Supplementary Figure 3 | Knockdown of USP8, but not of the other five DUBs, decreases the protein levels of trichoplein regardless of ciliogenesis and cell cycle. 24 h after transfection with control or IFT20 siRNA, RPE1 cells were further transfected with pooled siRNA for indicated DUBs and cultured for 48 h. Percentages of ciliated cells (**a**) and cyclin A-positive cells (**b**) are shown as mean ± SD from three or four independent biological replicates (n > 200 each). Immunoblotting of cyclin A (**b**), IFT20, GAPDH and trichoplein (**c**) are shown. It is notable that knockdown of USP8 (**c**; lane 10), but not of the other five DUBs, prominently decreased the trichoplein level in IFT20-depleted cells. **p<0.01, n.s., not significant, two-tailed unpaired student's *t*-tests.

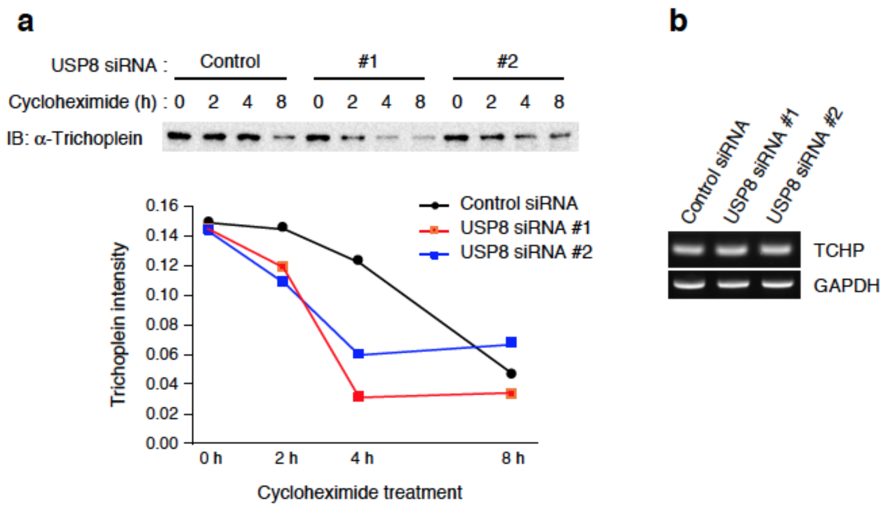

Supplementary Figure 4 | USP8 regulates the protein level of trichoplein but not the mRNA level. RPE1 cells were transfected with control or USP8 siRNA (#1 or #2) and cultured for 48 h in the presence of serum. (**a**) The cells were treated with 200 nM cycloheximide for the indicated times. To accurately evaluate the kinetics of trichoplein turnover in Figure 1d, the loading doses were adjusted so that the starting signals of anti-trichoplein immunoblot were identical. A graph shows arbitrary units of trichoplein intensities. (**b**) The cells were subjected to reverse transcription PCR.

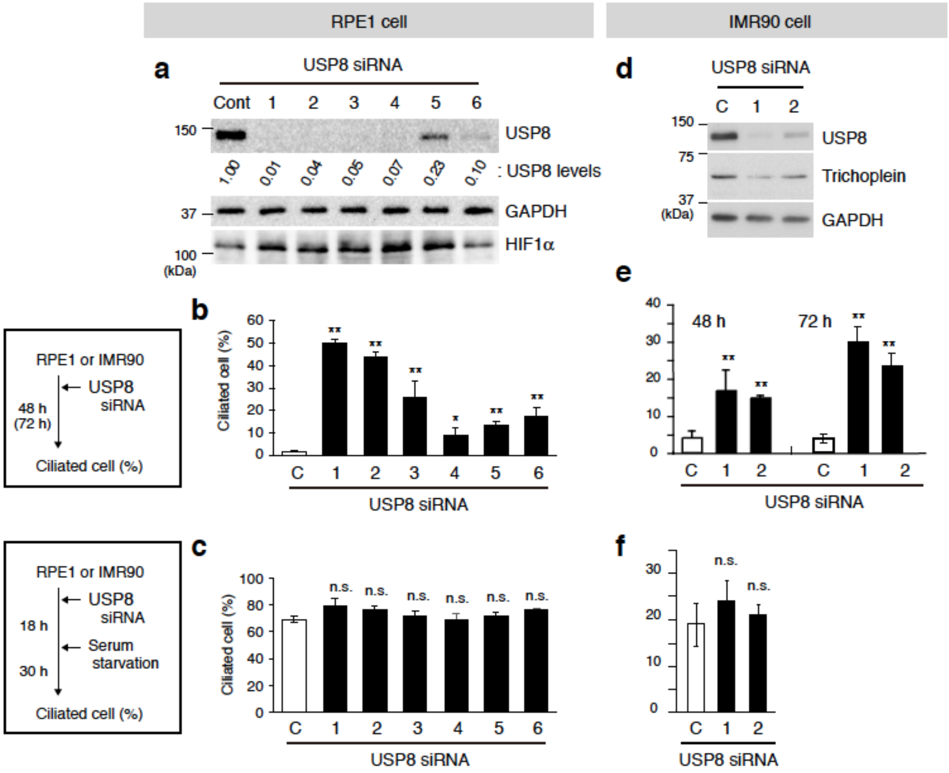

Supplementary Figure 5 | Validation of the effects of USP8 knockdown in RPE1 and IMR-90 cells. RPE1 (**a-c**) and IMR-90 cells (**d-f**) transfected with control or indicated USP8 siRNA (#1-6) were cultured in serum-supplemented normal medium (**a, b, d, e**) or subjected to serum starvation (**c, f**) according to the experimental procedures shown in left panels. Immunoblotting analysis (**a, d**) and percentages of ciliated cells (**b, c, e, f**; mean ± SD from three independent biological replicates, n > 200 each) are shown. Normalized USP8/GAPDH levels were evaluated by immunoblotting. **p<0.01, *0.01<P<0.05, n.s., not significant, two-tailed unpaired student's *t*-tests.

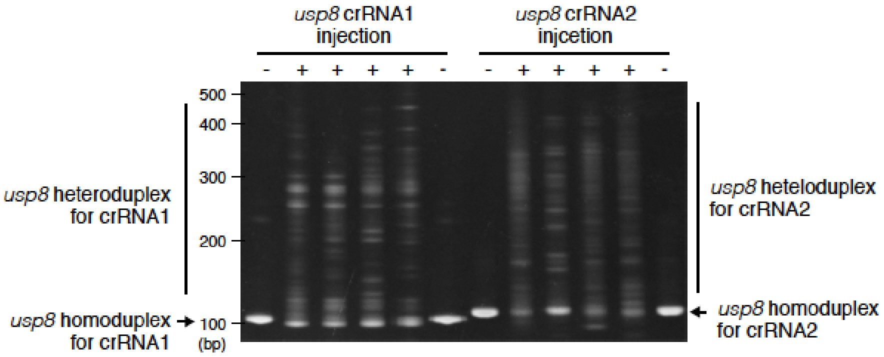

Supplementary Figure 6 | Knockout of usp8 in zebrafish. Heteroduplex mobility assay of PCR products amplifying the crRNA-targeted genomic region of usp8. The positions of the expected homoduplex are indicated by black arrows. The reductions of homoduplex and and the existence of multiple heteroduplexes, which exhibit the retarded mobility bands, indicates that the both usp8 crRNA1 and crRNA2 efficiently edited the usp8 genes, resulting in usp8-KO.

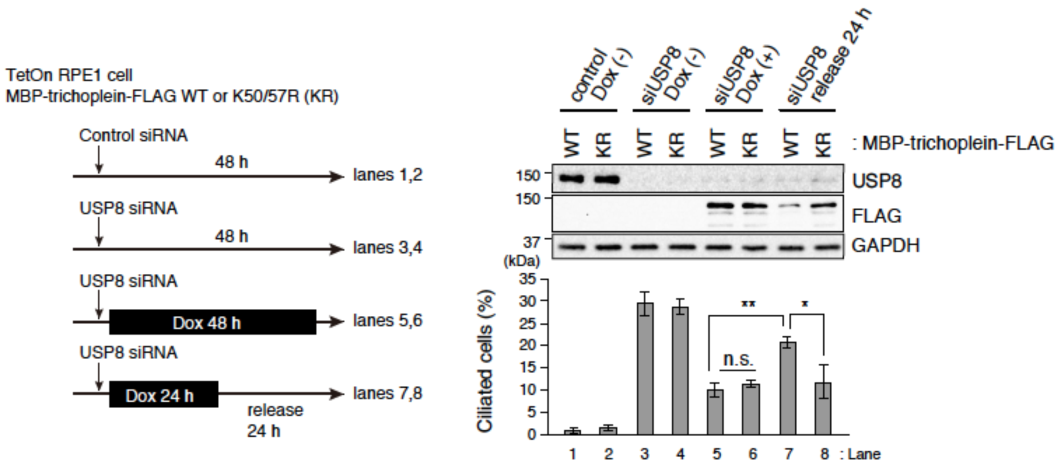

Supplementary Figure 7 | USP8 knockdown-mediated ciliogenesis depends on trichoplein degradation. TetOn-RPE1 cells that express MBP-trichoplein-FLAG WT or K50/57R (KR), a non-degradable mutant, were transfected with control (lanes 1, 2) or USP8 siRNA (#1) cells and cultured for 48 h in the absence (lanes 3, 4) or presence of 30 ng ml^{-1} of Dox (lanes 5, 6). Note that overexpression of WT and K50/57R comparably blocked the ciliogenesis in USP8-depleted cells (graph; lanes 5, 6). Release from the Dox treatment notably reduced the WT protein levels, but had only a marginal effect on the K50/57R levels (anti-FLAG blot; lanes 7, 8). In this situation, the ciliogenesis was prevented in the K50/57R cells but not in the WT cells (graph; lanes 7, 8), indicating that degradation of trichoplein is required for ciliogenesis in USP8-depleted cells. A graph represents percentages of ciliated cells (mean ± SD from three independent biological replicates, n > 200 each). **P<0.01, *0.01<P<0.05, n.s., not significant, two-tailed unpaired student's *t*-tests.

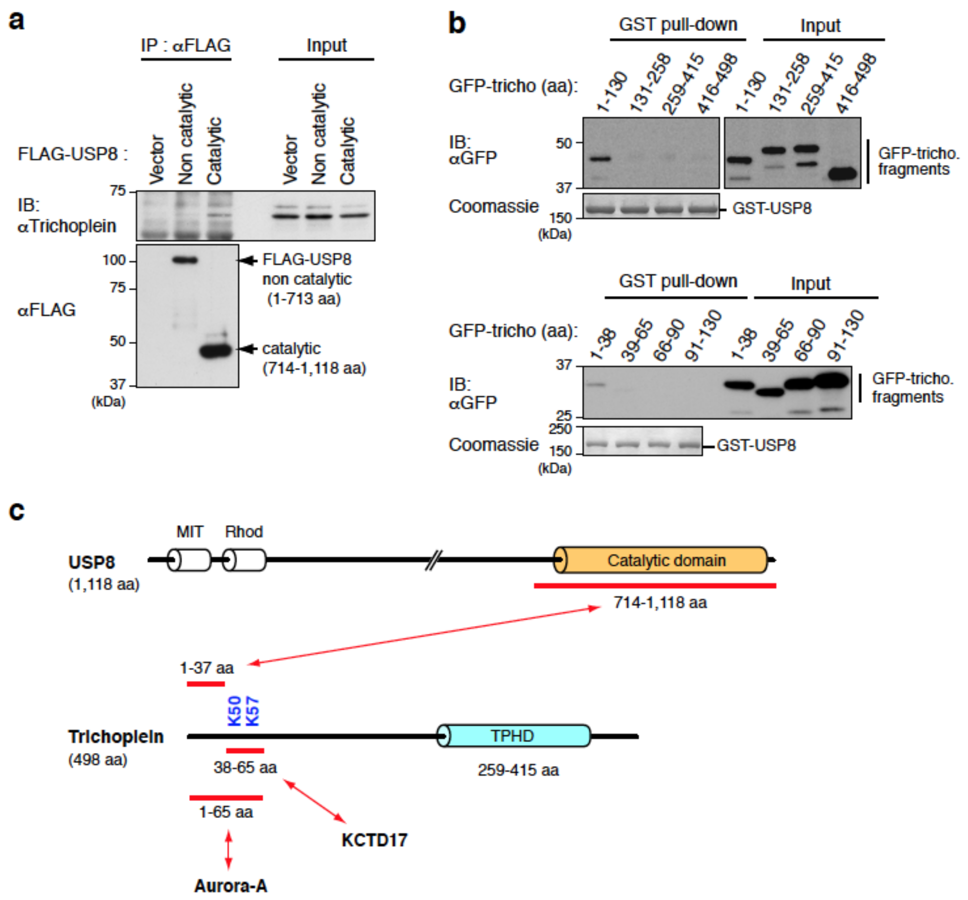

Supplementary Figure 8 | Interaction between trichoplein and USP8. (**a**) HEK293T cells were transfected with the amino-terminal non-catalytic region (1-713 aa) or the carboxyl-terminal catalytic region (714-1,118 aa) of the FLAG-USP8. Anti-FLAG immunoprecipitates were analyzed by immunoblotting with anti-trichoplein. (**b**) HEK293T cells transfected with indicated GFP-trichoplein fragments were subjected to GST pull-down assays using bacterially purified GST-USP8. (**c**) Summary of the interactions among indicated proteins. Trichoplein K50 and K57 are its polyubiquitination sites by CRL3^{KCTD17}.

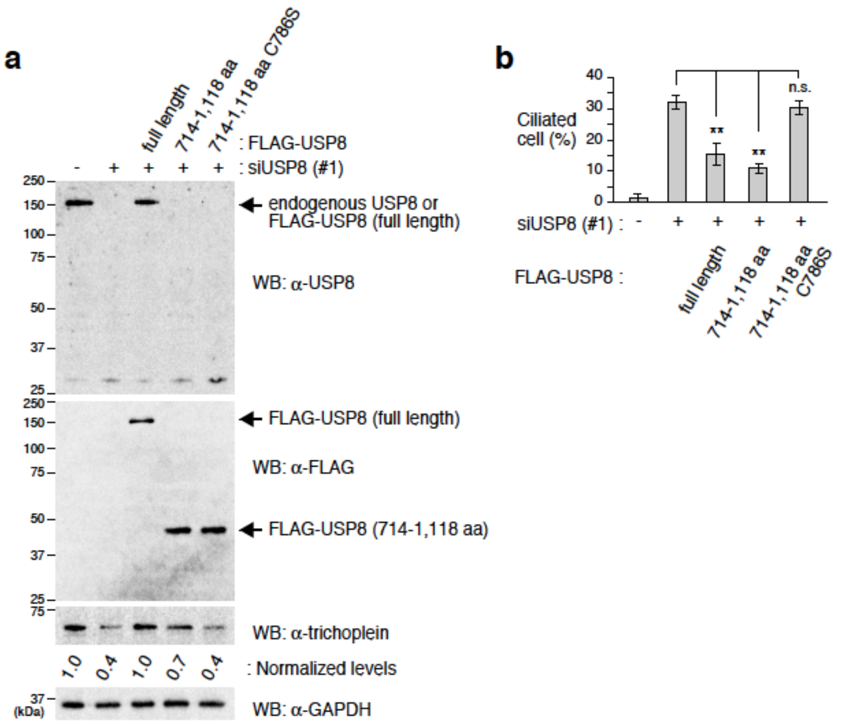

Supplementary Figure 9 | The catalytic domain of USP8 is sufficient for blocking ciliogenesis and trichoplein degradation Endogenous levels of expression of FLAG-USP8 (full length) and its 714-1,118 aa truncation mutant that mainly contained the catalytic domain were capable for blocking the trichoplein degradation and ciliogenesis of USP8-depleted RPE1 cells (USP8 siRNA #1). The inactive 714-1,118 aa truncation mutant (C786S) had no effect. (**a**) Immunoblotting analysis of USP8, FLAG, trichoplein and GAPDH are shown. Normalized trichoplein/GAPDH levels are shown as mean from three independent biological replicates. (**b**) Percentages of ciliated cells are shown as mean ± SD from three independent biological replicates (n > 200 each). **p<0.01, n.s., not significant, two-tailed unpaired student's *t*-tests.

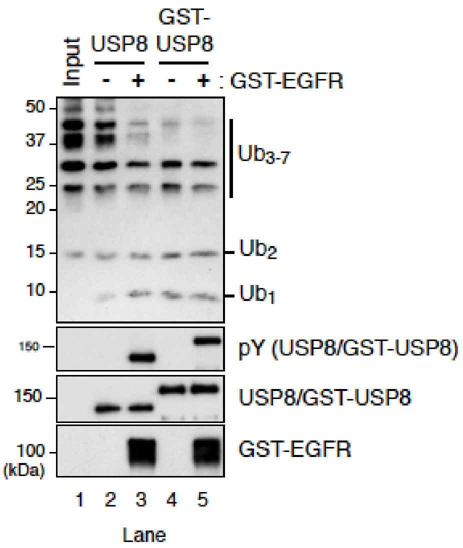

Supplementary Figure 10 | Non-tagged, but not GST-tagged, USP8 is catalytically activated by GST-EGFR-mediated phosphorylations *in vitro*. Recombinant, bacterially purified, non-tagged USP8 (USP8) or GST-USP8 was incubated with or without GST-EGFR (669-1,210 aa) for 15 min at 30°C, and then incubated with ubiquitin oligomers (Ub_{3-7}) for 15 min at 37 °C *in vitro*. Immunoblotting analysis of ubiquitin, phospho-tyrosine (pY), USP8 ad GST are shown, Ub_1, ubiquitin monomer; Ub_2, ubiquitin dimmer.

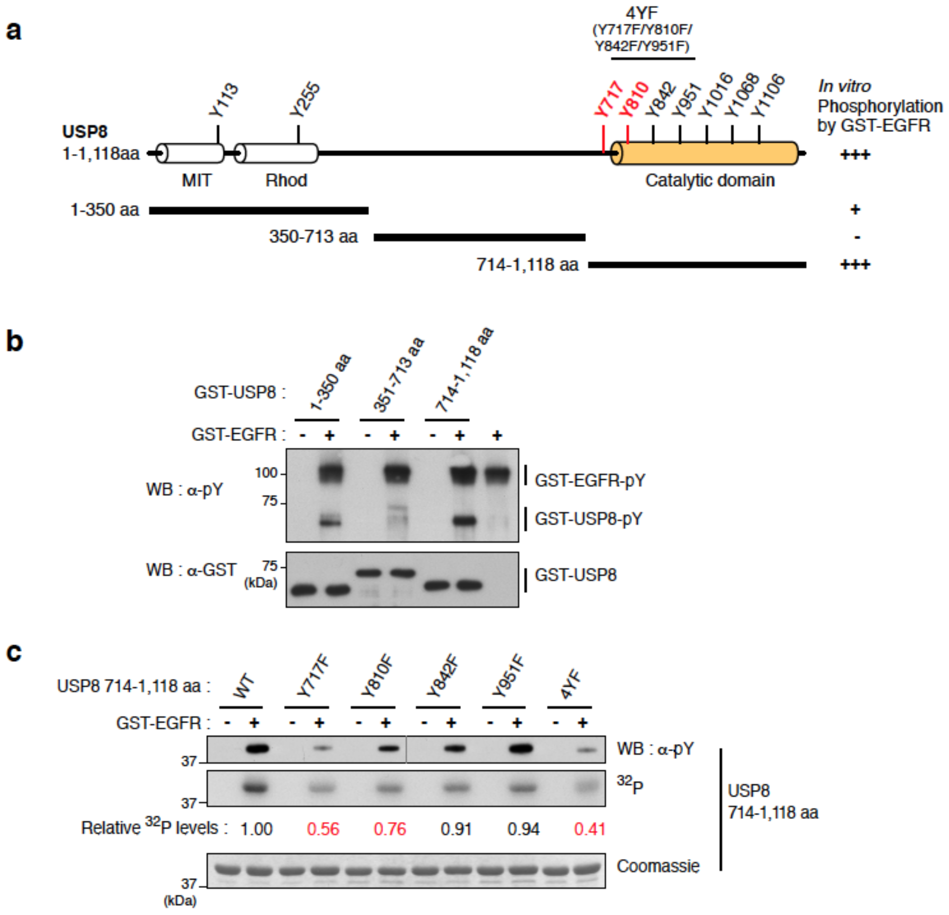

Supplementary Figure 11 | EGFR phosphorylates USP8 on Tyr-717 and Tyr-810 *in vitro*.
(**a**) Summary of the results from *in vitro* phosphorylation assays. (**b**) Recombinant, bacterially purified, GST-USP8 proteins (1-350, 351-713 or 714-1,118 aa) were subjected to *in vitro* phosphorylation assays GST-EGFR (669-1,210 aa), and subjected to immunoblotting analysis with anti-phospho-tyrosine (pY) and anti-GST antibodies. (**c**) Recombinant, bacterially purified, non-tagged USP8 (714-1,118 aa) proteins were incubated with GST-EGFR (669-1,210 aa) *in vitro*, and then subjected to anti-phospho-tyrosine immunoblotting. Incorporated ^{32}P levels shown as mean from three independent biological replicates were evaluated by autoradiography.

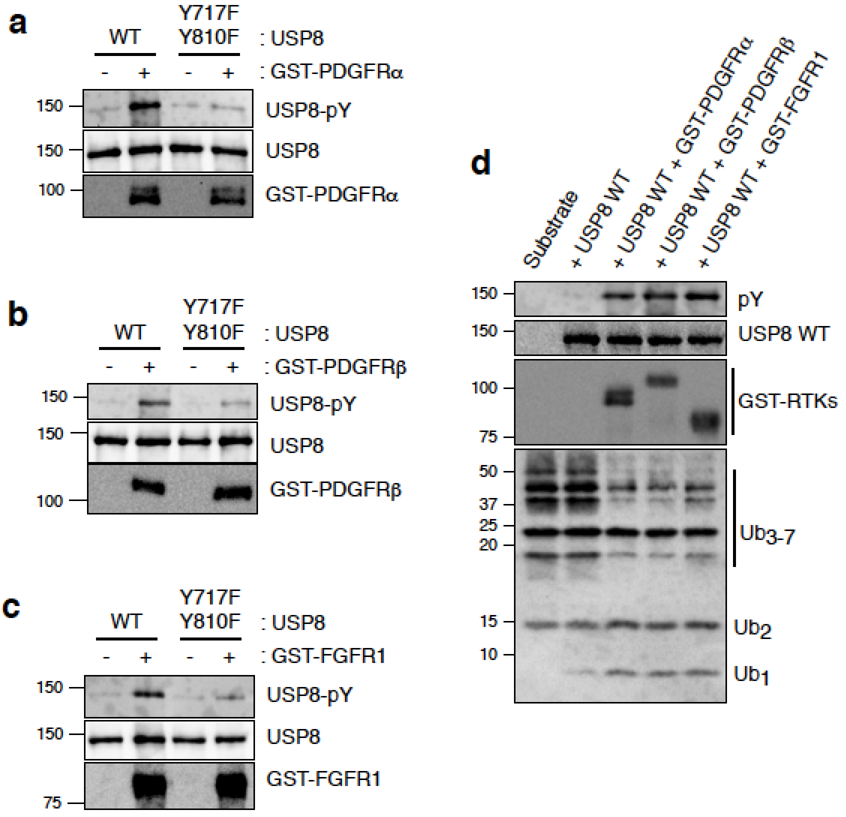

Supplementary Figure 12 | Effects of PDGFRs- or FGFR1-mediated phosphorylation of USP8 on its DUB activity. a-c, Recombinant, bacterially purified, non-tagged USP8 (USP8) WT or Y717F/Y810F was treated with or without purified GST-PDGFRα (**a**), GST-PDGFRβ (**b**) or GST-FGFR1 (**c**) for 15 min at 30°C *in vitro*, and then analyzed by immunoblotting. (**d**) Non-phosphorylated and phosphorylated USP8 WT were prepared as described in **a-c**, and then incubated with ubiquitin oligomers (Ub_{3-7}) for 15 min at 37 °C. Ub_1, ubiquitin monomer; Ub_2, ubiquitin dimmer.

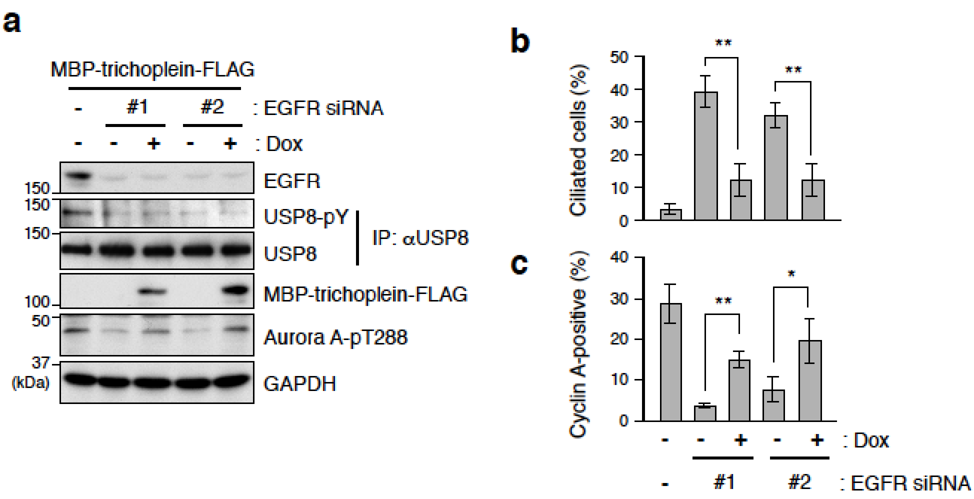

Supplementary Figure 13 | Effects of MBP-trichoplein-FLAG expression in EGFR-depleted cells. TetOn-RPE1 MBP-trichoplein-FLAG cells were transfected with EGFR siRNA (#1 or #2). 6 h after transfection, the cells were treated with doxycycline (Dox; 30 ng ml^{-1}) for 42 h to induce the expression MBP-trichoplein-FLAG. Immunoblotting analysis (**a**), percentages of ciliated cells (**b**) and cyclin A-positive cells (**c**) are shown. Graphs represent mean ± SD from three independent biological replicates (n > 200 each). **p<0.01, *0.01<P<0.05, n.s., not significant, two-tailed unpaired student's *t*-tests.

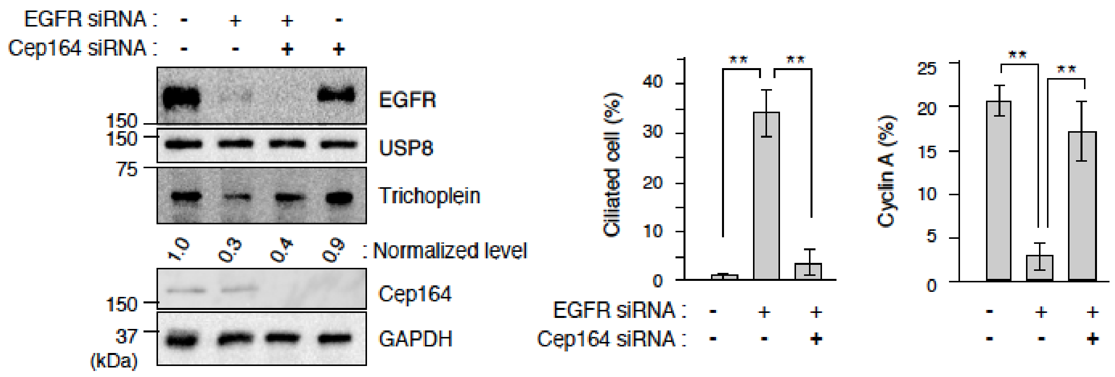

Supplementary Figure 14 | EGFR knockdown in Cep164-depleted RPE1 cells. 24 h after transfection with Cep164 siRNA, RPE1 cells were further transfected with EGFR siRNA (#1), and cultured for 48 h. Immunoblotting analysis, percentages of ciliated cells and cyclin A-positive cells are shown. Graphs represent mean ± SD from three independent biological replicates (n > 200 each). **p<0.01, n.s., not significant, two-tailed unpaired student's *t*-tests.

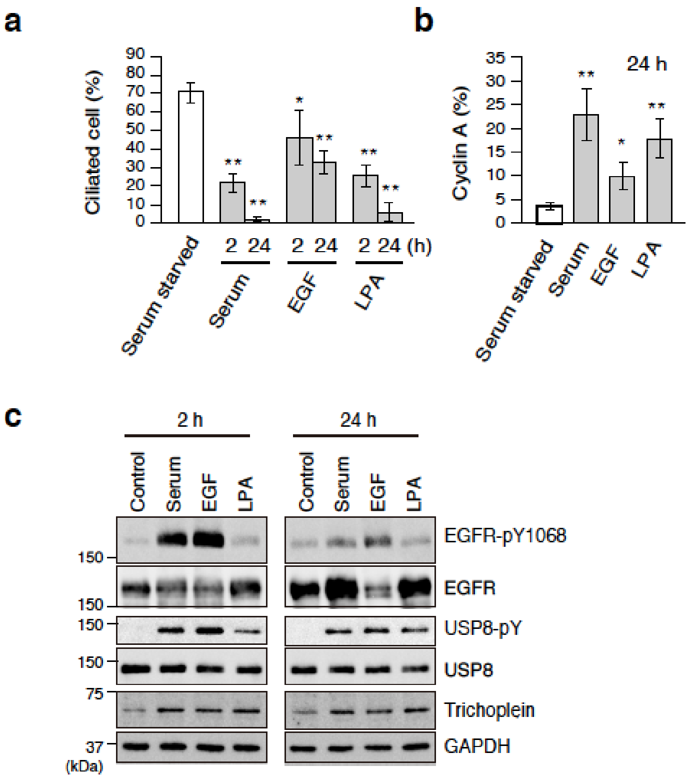

Supplementary Figure 15 | Activation of EGFR-USP8-trichoplein pathway in serum-induced ciliary disassembly. 48 h after serum starvation, RPE1 cells were treated with serum (10% FBS), EGF (10 ng ml^{-1}) or LPA (10 μM) for 2 or 24 h. Percentages of ciliated cells (**a**) and cyclin A-positive cells (**b**) are shown as mean ± SD from three independent biological replicates (n > 200 each). (**c**) Immublotting analysis with indicated antibodies are shown. **p<0.01, *0.01<p*<0.05, n.s., not significant, two-tailed unpaired student's *t*-tests.

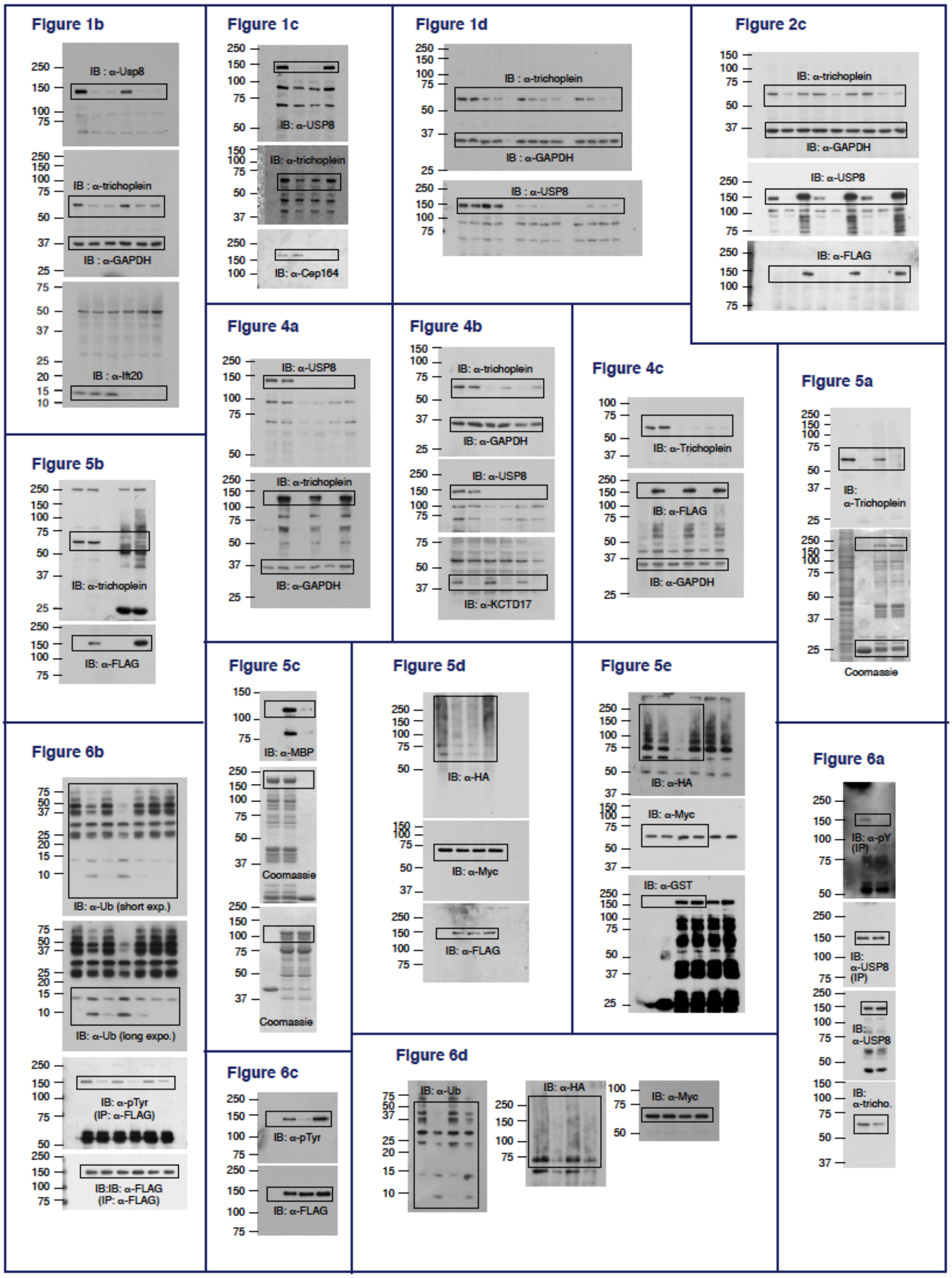

Supplementary Figure 16 | Uncropped versions of immunoblot data.

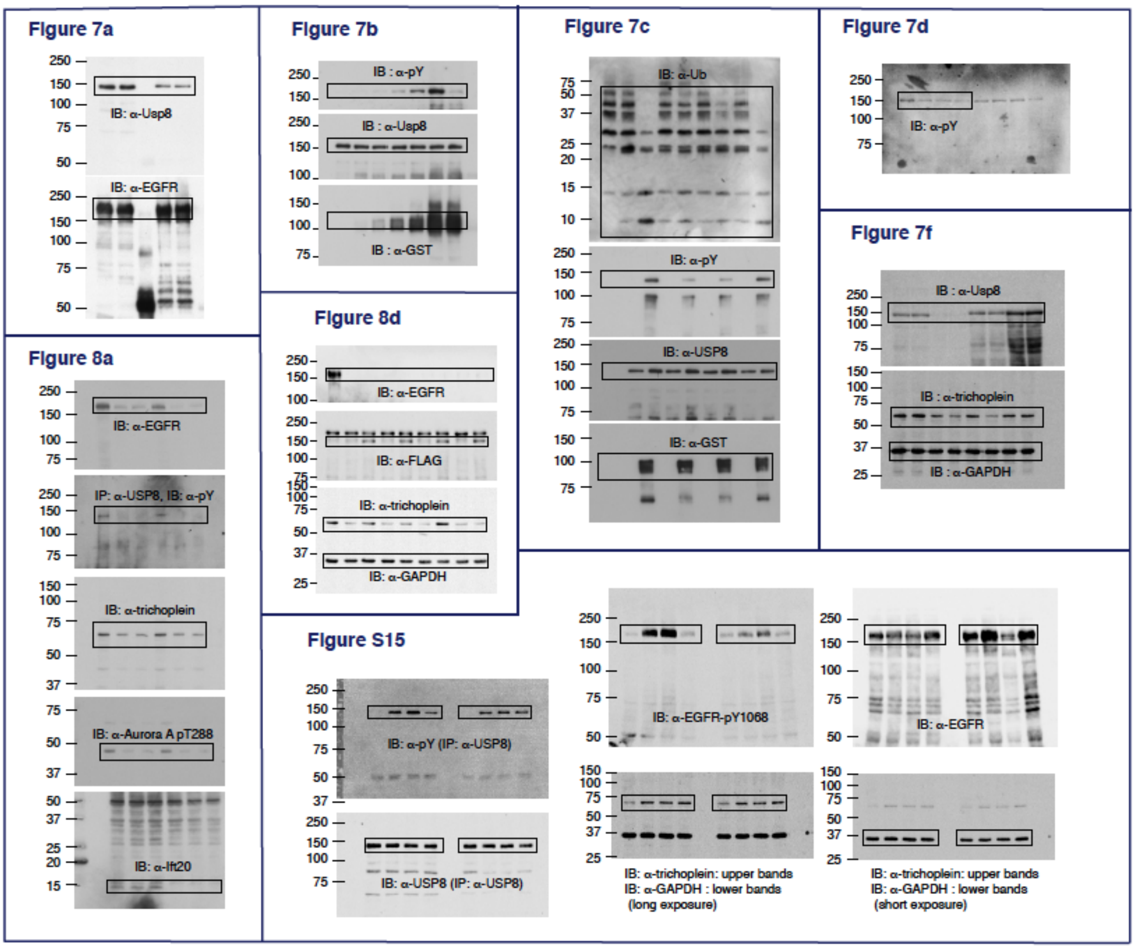

Supplementary Figure 16 (continued) | Uncropped versions of immunoblot data.

Uncropped versions of most important immunoblotting data are shown.

Supplementary Table 1 | siRNA sequences

**Name**	**Target sequense**
USP8 siRNA #1	AAGGCTCGTATTCATGCAGAA
USP8 siRNA #2	AACCACTAGCATCCACAAGTA
USP8 siRNA #3	CAGGGTCAATTCAAATCTACA
USP8 siRNA #4	GGCAAGCCATTTAAGATTA
USP8 siRNA #5	CTTCGTAACTTAGGAAATA
USP8 siRNA #6	CAGGTTCAGGCAAGCCATTTA
TCHP siRNA #1	AAGGCAGAATGGAGCTCTAAA
TCHP siRNA #2	TCCCAGCGCATTCCTTTGCAA
AURKA siRNA #1	TCCCAGCGCATTCCTTTGCAA
AURKA siRNA #2	CAGGGCTGCCATATAACCTGA
KCTD17 siRNA	CACGCAAATGGTCTCCACCAT
EGFR siRNA #1	ATAGGTATTGGTGAATTTAAA
EGFR siRNA #2	CAGGAACTGGATATTCTGAAA
IFT20 siRNA	CAGAAAAYAGTTGGTGGTTTA
Cep164 siRNA	CAGAGTGAAGGTGTATCGCTT
Control siRNA	AATTCTCCGAACGTGTCACGT

Supplementary Table 2 | crRNA, tracrRNA, and PCR primers

Name	Sequense (5'-3')
usp8_crRNA1	guucagaucuccgagugacgguuuuagagcuaugcuguuuug
usp8_crRNA2	ggaggugcggaagaagcuggguuuuagagcuaugcuguuuug
tracRNA	aaacagcauagcaaguuaaaauaaggcuaguccguuaucaacuugaaaaagugg caccgagucggugcu
usp8_gF1	atgagctgaagatgccgtcc
usp8_gR1	tgctcttgtccggcttgatt
usp8_gF2	tccagcgctgtgttcttctc
usp8_gR2	cttctccgttcgctcctctc
